# Case report: The first account of undifferentiated sarcoma with epithelioid features originating in the pleura

**DOI:** 10.3389/fmed.2024.1301941

**Published:** 2024-02-01

**Authors:** Ling-Xi Xiao, Li Liu, Wang Deng

**Affiliations:** ^1^Department of Respiratory and Critical Care Medicine, Second Affiliated Hospital of Chongqing Medical University, Chongqing, China; ^2^Department of Pathology, Second Affiliated Hospital of Chongqing Medical University, Chongqing, China

**Keywords:** undifferentiated sarcoma, epithelioid features, pleural tumors, immunohistochemistry, TP53 genes

## Abstract

Undifferentiated epithelioid sarcoma (USEF) is a rare subtype of undifferentiated soft tissue sarcoma that presents unique challenges in clinical diagnosis and treatment. Here, we report a case of USEF occurring in the pleura of a 51-year-old man for the first time. Thoracoscopic examination revealed widespread nodular changes, and pathological analysis confirmed the presence of numerous epithelioid atypical cells. Immunohistochemical (IHC) analysis demonstrated an undifferentiated phenotype with distinct characteristics: epithelial membrane antigen (foci +), vimentin (+), Ki-67 (+70% +), TTF-1 (+), P53 (mutant type +90%), INI-1 (+), and CK5/6 (small foci +). Immunohistochemical examination of the tumor showed that the tumor was an undifferentiated epithelioid sarcoma. High-throughput DNA sequencing revealed pivotal mutations, including a nonsense mutation in the *NF1* gene (c.641A > G(p.H214R)). and critical *TP53* missense mutation (c.641A > G(p.H214R)). This *TP53* mutation, with a tumor mutation burden of 16.5 Muts/Mb, signifies a high level of genomic instability, likely contributing to the rapid progression and aggressiveness of the disease. Detection of the *TP53* mutation provides essential insights, indicating the disease’s rapid progression and highlighting the potential for targeted therapies. Although the patient’s disease progressed extremely rapidly and he tragically died within a week, we discussed the results of IHC and DNA sequencing in detail and discussed his possible treatment options. Insights gained from this case will be critical in shaping future diagnostic and therapeutic paradigms for USEF, particularly in the context of TP53 mutations.

## 1 Introduction

Undifferentiated soft tissue sarcoma (USTS) constitutes a rare and heterogeneous group of soft tissue cancers that pose challenges for their effective differentiation using current diagnostic tools. Undifferentiated pleomorphic sarcoma, undifferentiated spindle cell sarcoma, undifferentiated round cell sarcoma, and undifferentiated epithelioid sarcoma (USEF) are among the subtypes of USTS (1). However, the existing literature on USEF is notably sparse.

USEF is commonly identified in older age groups, displaying no gender bias and often presenting with a larger size at diagnosis. It frequently occurs centrally, such as in the proximal thigh and torso (1). Traditional treatment methods for USEF, including surgical resection, chemotherapy, and radiotherapy, present significant challenges due to the biological complexity and varied treatment responses of USEF, resulting in a generally poor prognosis (2).

Due to limited research, the understanding of USTS and their anticipated outcomes remains insufficient. Among adults, undifferentiated pleomorphic sarcoma (UPS) originating from the limbs or trunk has been reported to have a 5-year metastasis-free survival rate of 83% (3). However, USEF appears to exhibit higher aggressiveness (2). highlighting the importance of discussing potential therapeutic approaches.

In this report, we aim to provide a comprehensive and detailed description of a unique case involving a pleural tumor presenting as USEF, contributing to an enhanced understanding of this extremely rare condition.

## 2 Case description

A 51-year-old man was admitted to the Department of Respiratory and Critical Care Medicine on March 31, 2023, with a 5-month history of cough and sputum and recent exacerbation of shortness of breath, wheezing, and fatigue lasting for 3 days. He had a 20-year history of smoking (approximately 20 cigarettes per day) and alcohol consumption (approximately 200 mL per day) and no known family history of cancer, genetic diseases, psychiatric disorders, nor a history of long-term medication use. Additionally, there was no evidence of current or previous soft tissue tumors or history of radiation exposure. Physical examination revealed diminished breath sounds in the right lung with solid percussion.

**Figure 1:**
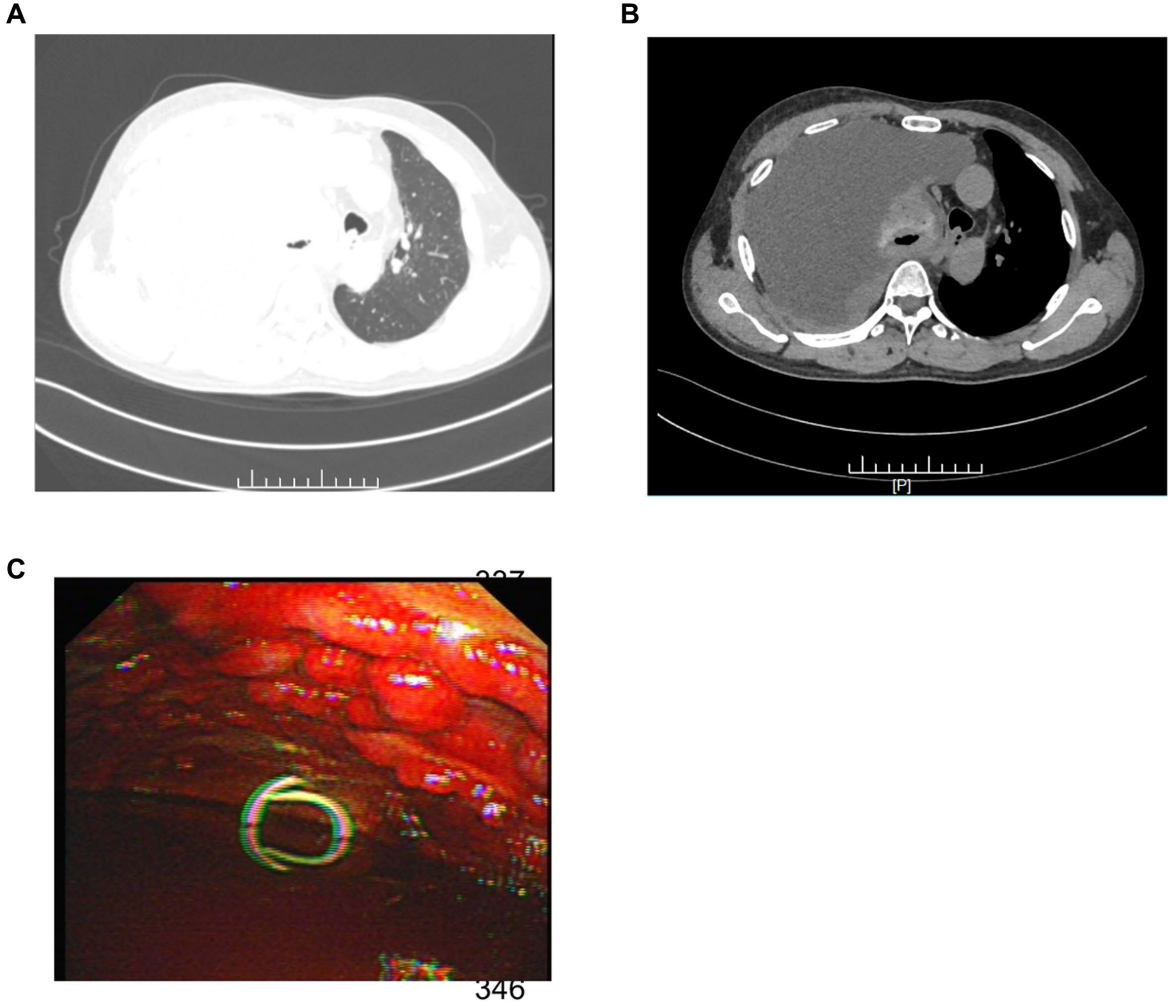
**(A)** The patient’s chest CT (lung window) exhibited extensive right pleural effusion. **(B)** The mediastinal window of the chest CT revealed significant right lung compression and irregular pleural thickening. **(C)** Thoracoscopy exposed diffuse nodular changes of diverse sizes in the parietal pleura along with substantial hemorrhagic effusion in the pleural cavity.

Upon admission, the patient’s complete blood work showed abnormal values. Specifically, the CRP was significantly high at 69.84 mg/L (<10 mg/L), and the leukocyte count was elevated at 16.88 × 10^9/L (3.50–9.50 × 10^9/L). Chest computed tomography (CT) demonstrated substantial right pleural effusion, causing significant compression of the right lung, and irregular thickening of the right pleura, measuring approximately 14.7 mm at its thickest point (Figures 1A,B), with no evidence of lymph node or visceral dissemination at presentation. Subsequent thoracentesis was performed, and the pleural fluid, appearing hemorrhagic, was identified as an exudate. Biochemical analysis indicated high lactate dehydrogenase levels.

Immunohistochemical (IHC) analysis of the pleural effusion sample further revealed cytokeratin (CK) (foci +), vimentin (+), calretinin (−), MC (−), D2–40 (−), CK5/6 (−), TTF − 1 (+), NapsinA (−), P63 (−), CK7 (foci +), Ki-67 (+30%+), and WT-1 (−). This comprehensive analysis played a crucial role in elucidating the underlying pathology and guiding subsequent steps in patient management. Images of immunohistochemical staining are shown in Supplementary Figure S1.

**Figure 2:**
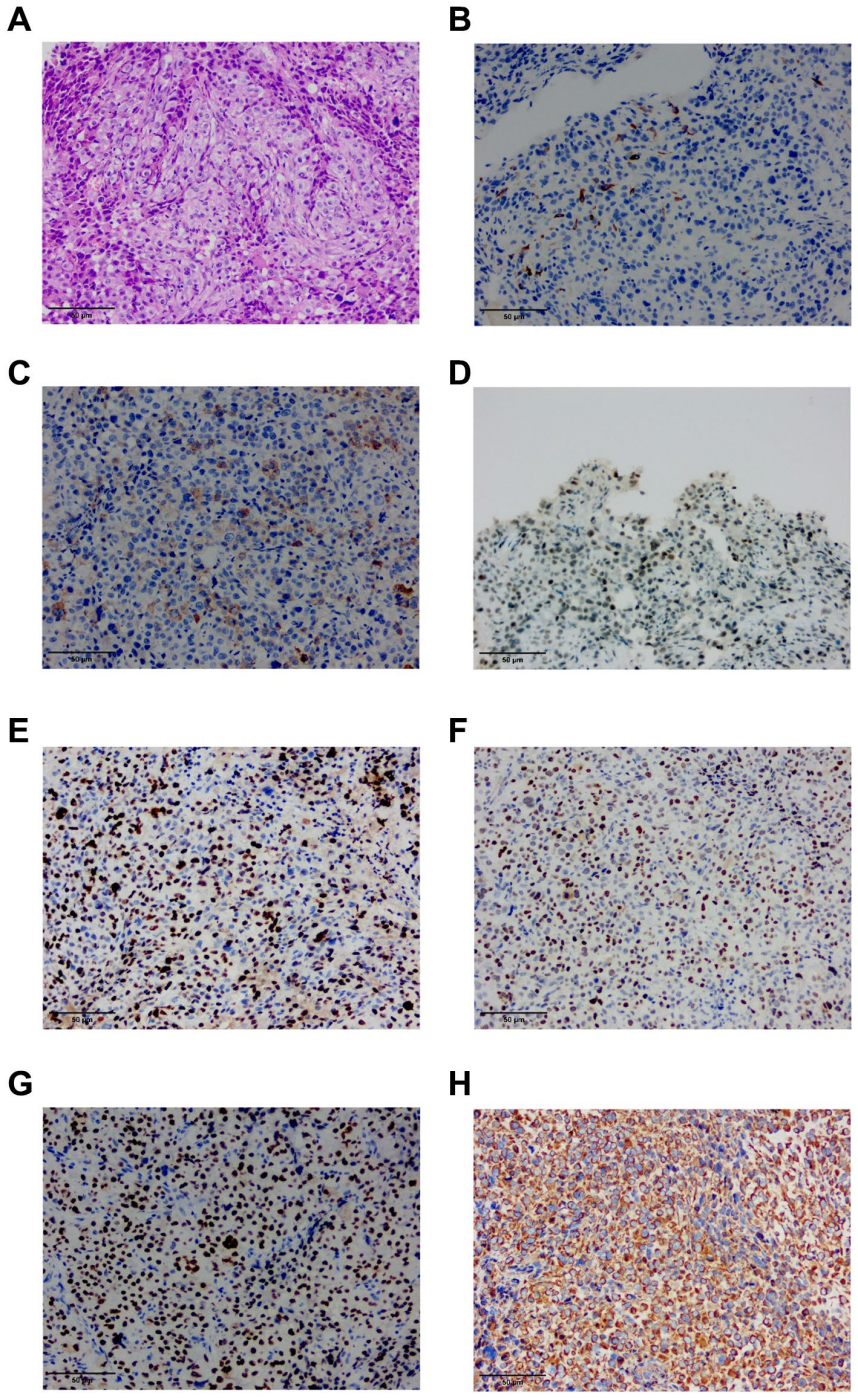
**(A)** HE staining finding at 20x magnification of the ultrasound-guided endoscopic fine-needle aspiration (USEF) sample from the right parietal pleura is shown. **(B)** Immunohistochemical staining for CK5/6 shows positive staining in small foci. **(C)** Immunohistochemical staining for EMA demonstrates positive staining in the lesion. **(D)** Immunohistochemical staining for INI-1 shows positive results. **(E)** Immunohistochemical staining for Ki-67 shows a high proliferation index with >70% positivity. **(F)** Immunohistochemical staining for P53 shows a mutant type with approximately 90% positive staining. **(G)** Immunohistochemical staining for TTF-1 indicates positive results. **(H)** Immunohistochemical staining for vimentin (Vim) shows positive staining.

The patient underwent a thoracoscopic biopsy, which revealed a significant amount of hemorrhagic pleural effusion within the pleural cavity. The pleural wall exhibited diffuse nodular lesions of varying sizes (Figure 1C). The pathological biopsy revealed a grayish-white color on the cut surface. Microscopic examination of sections prepared from the surgical specimen displayed extensive areas filled with epithelioid atypical cells. These cells were large and had abundant cytoplasm, and some areas showed eosinophilic staining. The nuclei were large, with a high nucleo-cytoplasmic ratio. In some tumor cells, nucleoli were visible, and pathological nuclear division was apparent (Figure 2A).

IHC analysis of the pleural nodules revealed the following results: epithelial membrane antigen (EMA) (foci +), Vim (+), Ki-67 (+70% +), TTF-1 (+), P53 (mutant type +90%), P40(−), calretinin (−), INI-1 (+), CK5/6 (small foci +), WT-1 (−), NapsinA (−), and CK7 (−). The immunohistochemical phenotype was undifferentiated, complicating the determination of the origin of tumor cells (Figures 2B–H).

**Figure 3:**
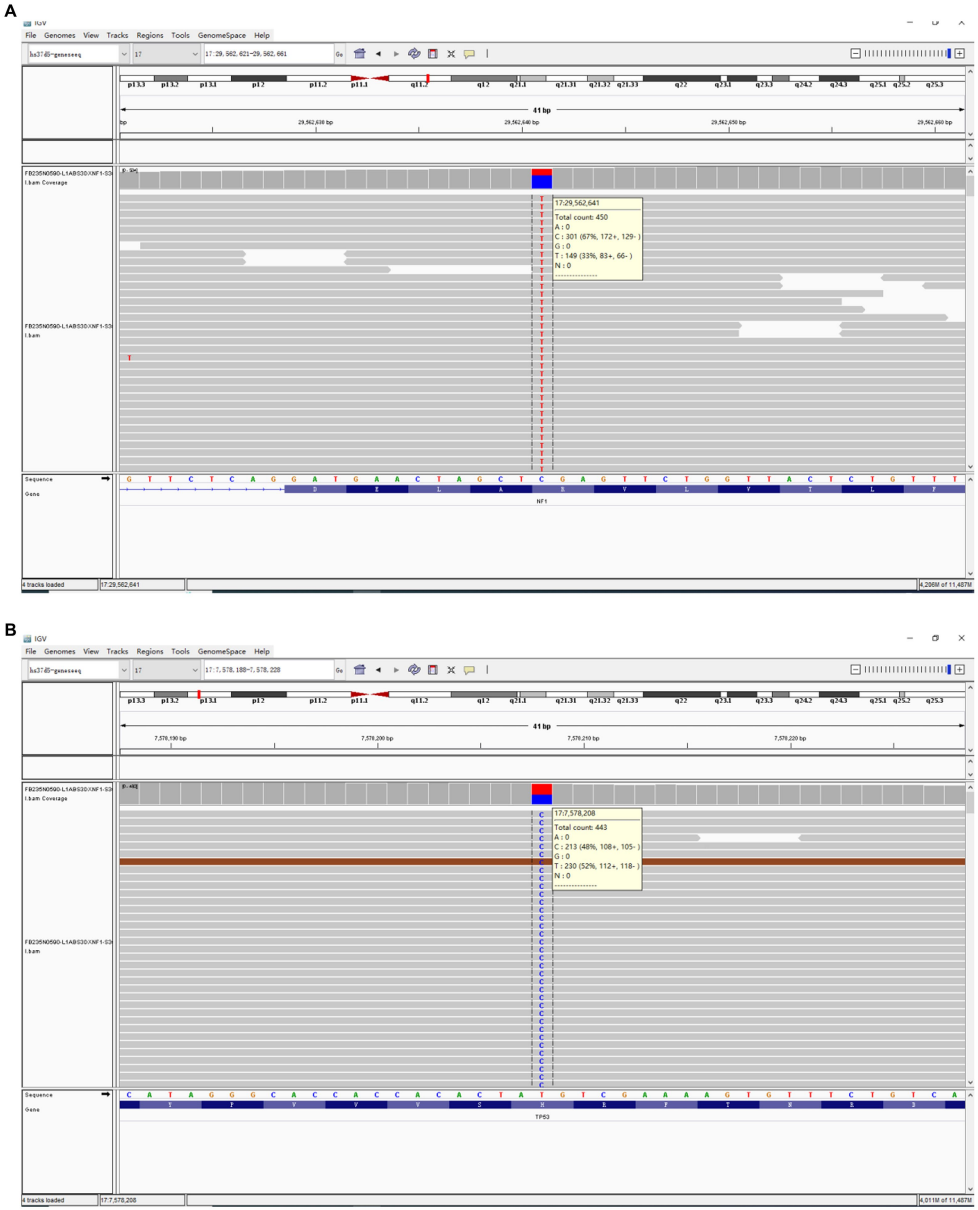
The comprehensive genomic profiling based on Next-Generation Sequencing has revealed a nonsense mutation in the NF1 gene (c.3721C > T(p.R1241*)) **(A)** within the USEF component and a missense mutation in the TP53 gene (c.641A > G(p.H214R)) **(B)**.

Next,-Generation Sequencing covered exons, fusion-related introns, variable splicing regions, and specific microsatellite (MS) site areas of 506 genes associated with soft tissue sarcoma typing and mutations, totaling approximately 1.73-Mb base positions. Next-Generation Sequencing (NGS) revealed two significant mutations: a nonsense mutation in the NF1 gene, c.3721C > T(p.R1241*), depicted in Figure 3A, and a missense mutation in the TP53 gene, c.641A > G(p.H214R), illustrated in Figure 3B. The abundance and specific details of these mutations are summarized in Table 1. For comprehensive data on additional gene mutations identified in this study, please refer to Supplementary Table S1. Additionally, a high tumor mutation burden (TMB) of 16.5 Muts/Mb was noted. These findings related to *NF1* and *TP53* are significant as they could influence the tumor’s characteristics and potential treatment strategies. Additionally, MS instability–high (MSI-H) status was not detected, and HLA-I typing was assessed as partially pure. However, these findings did not clarify the origin of the tumor cells.

**Table 1:** Main mutation frequencies of unclassified sarcoma with epithelioid features.

Gene	Mutation	Alteration	Variant allele fraction
NF1	p.R1241* exon 28 nonsense mutation	c.3721C > T(p.R1241*)	32.99%
TP53	p.H214R exon 6 missense mutation	c.641A > G(p.H214R)	48.14%

**Figure 4:**
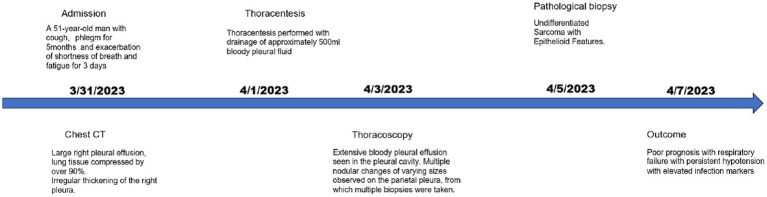
Timeline scheme of the major clinical event of the patient.

Considering the unfavorable prognosis and high treatment costs, the patient chose against aggressive therapies, such as radiotherapy, chemotherapy, and surgery. Instead, the patient received targeted supportive care. However, the patient’s condition rapidly deteriorated. Within a week, he developed respiratory failure with persistent hypotension, unresponsive to vasopressors. This was accompanied by significantly elevated infection markers: leukocyte count leukocyte count was 24.61 × 10^9/L (3.50–9.50 × 10^9/L), Procalcitonin (PCT) level was 1.2798 ng/mL(0.0000–0.0500 ng/mL), and CRP was >200 mg/L (<10 mg/L). Due to these circumstances, the family decided to cease all treatments, and the patient unfortunately died on April 7, 2023 (Figure 4).

## 3 Immunohistochemistry and next-generation sequencing techniques

IHC was performed in the Department of Pathology of the Second Affiliated Hospital of Chongqing Medical University. Initially, the slides undergo dewaxing and hydration. Subsequently, they are incubated in a 3% hydrogen peroxide solution for 10 min to block endogenous peroxidase activity. This step is followed by washing the slides three times with phosphate-buffered saline (PBS) at pH 7, each time for 3 min. The slides are then placed in a citrate buffer solution (pH 6.0) and subjected to microwave heating for antigen retrieval, twice, each time for 10 min. After heating, the slides are allowed to cool at room temperature. Subsequently, they are again washed three times with PBS at pH 7.4, each for 3 min. Depending on the tissue size, an appropriate amount of primary antibody is applied and incubated overnight at 4°C. After incubation, the slides are washed three times with PBS, each for 3 min. This is followed by the addition of an appropriate amount of enzyme-labeled polymer goat anti-mouse/rabbit IgG; thereafter, the slides undergo incubation at room temperature for 20 min. After incubation, the slides are washed three times with PBS, each for 3 min. Finally, the slides are visualized using a freshly prepared diaminobenzidine chromogen solution. Post-visualization, the slides are rinsed with PBS and counterstained with hematoxylin. We added positive and negative controls during the experiment to demonstrate the validity and reliability of the staining process, as seen in Supplementary Figure S2. IHC results are evaluated and interpreted by independent pathologists.

Next,-Generation Sequencing (NGS) was performed employing the GENESEEQ PRIME^™^ platform (Geneseeq Technology Inc., Nanjing, China). The process encompassed the following steps: Nucleic Acid Extraction: Formalin-Fixed Paraffin-Embedded (FFPE) nucleic acids were extracted using the FFPE Nucleic Acid Extraction Kit (Geneseeq Medical Device and Diagnostic Inc., Nanjing, China). Library Preparation: the library was constructed using the EGFR/ALK/ROS1/BRAF/KRAS/HER2 Gene Mutation Detection Kit (Geneseeq Medical Device and Diagnostic Inc., Nanjing, China). The method utilized for sequencing was the reversible terminator-based method, which offers high accuracy and read lengths. DNA Quantification and Purity Assessment: DNA concentration in the prepared samples was quantified using the highly sensitive Qubit^®^ 3.0 Fluorometer along with the Qubit^®^ DNA Assay Kit (Thermo Fisher Scientific Inc., Waltham, MA, United States). The purity of the DNA was assessed using the NanoDrop^™^ spectrophotometer (Thermo Fisher Scientific Inc., Waltham, MA, United States).

## 4 Discussion

Due to the limited ongoing research on USEF, both identification and treatment pose significant challenges. Treating USEF is notably difficult due to the scarcity of prospective studies and reliance on case reports within the academic community. In the present case, the patient initially exhibited symptoms of cough, sputum production, wheezing, and fatigue, leading to hospitalization. Throughout the diagnostic process, we utilized various methods, including chest CT, thoracoscopic pleural biopsy, immunocytochemical analysis (ICC), IHC, and genetic testing. Among these methods, ICC analysis is a rapid, minimally invasive, and cost-effective diagnostic tool, offering lower invasiveness, reduced complication risks, lower costs, and faster results than tissue biopsy. This approach proves particularly effective for differential diagnosis in patients with pleural effusion. Additionally, the utilization of positron emission tomography–CT scans holds significant value in such cases, allowing for the assessment of tumor response to treatment before and after therapy.

According to the 2020 WHO Classification of Soft Tissue and Bone Tumours, USTS can broadly be categorized into pleomorphic, spindle cell, round cell, and epithelioid subgroups (4). However, they lack specific defining features other than the absence of recognizable differentiation. Among these, UPS represents the largest group, with USEF being less extensively studied. The diagnostic criteria for USEF include epithelioid cell morphology and the absence of any immunohistochemical feature of specific differentials, demonstrating the absence of distinctive molecular aberration.

USEF represents a heterogeneous group of tumors that share certain pathological and immunohistochemical characteristics with epithelioid sarcoma (ES). However, these tumors do not meet the criteria for a definitive ES diagnosis (2). Studies have indicated that CK and EMA are more frequently present in ES, with a majority of ES cases exhibiting a loss of INI-1, as demonstrated using IHC (5). In contrast, every USEF sample expresses at least one epithelial marker (pan-CK and/or EMA). Unlike ES, USEF usually does not express CD34, and the absence of INI-1 and expression of CA-125 are less common (2). In this particular case, locally positive EMA and positive INI-1 were observed.

USEF is typically diagnosed by exclusion. MPM serves as a significant differential diagnosis based on radiological features. The presence of CK5/6 might suggest an MPM phenotype, but the absence of WT-1, calretinin, and D2-40, which are typically positive in MPM, reduces the likelihood of this condition (6). Additionally, although this case included an examination of pleural effusion cytology, it failed to identify MPM-associated markers, such as MTAP, 5-hmC, GLUT1, IMP3, and EZH2 (7).

From a clinical incidence perspective, differentiating pleural tumors from pleural metastasis caused by primary lung cancer is crucial (8). The presence of TTF-1 and Vim, which can also be seen in some cases of lung adenocarcinoma, suggests this possibility (9). However, the absence of CK7 and NapsinA further rules out the likelihood of lung adenocarcinoma (10).

After eliminating common tumors, it is crucial to consider and exclude rare conditions such as SMARCA4-deficient thoracic sarcoma. This recently discovered and uncommon condition is suggested by the presence of TTF-1, EMA, CK, INI-1, and Vim. However, the final next-generation sequencing test ruled out the possibility by not detecting the SMARCA4 inactivating mutation (11, 12).

In this study, the expression rate of Ki-67 in patient lesions exceeded 70%, signifying a significant predictor of poor prognosis. Ki-67, a nuclear protein associated with cellular proliferation, has elevated expression levels closely correlated with worsened clinical outcomes, especially in patients with high-risk soft tissue sarcomas (STS) (13). A high Ki-67 proliferation index has been established as a critical factor affecting the prognosis of patients with STS. A prospective study has indicated that using a Ki-67 grading system for assessing STS malignancy is more effective and reproducible than the traditional system adopted by the French Federation of Cancer Centers Sarcoma Group (FNCLCC) (14).

Finally, the diagnosis of USEF is made based on the absence of lineage-specific markers or corresponding fusion and mutation genes, as well as considering its histological appearance as epithelioid. Understanding this rare, the diagnosis of highly invasive soft tissue tumor requires careful examination of available literature. Reported cases of USEF, excluding occasional cases reported in non-English literature, have been summarized in Table 2.

**Table 2:** Previously reported cases of unclassified sarcoma with epithelioid features.

Case number	Author	Sex/age, y	Anatomical site	Initial therapy	Immunohistochemical findings	Genetic analysis	Outcome (duration of follow up)
1 (33)	Lang Y	F/66	Left lower leg	Surgical resection	Positive for vimentin, TFE3, CD68 and CD34	TFE3 gene amplification	No evidence of disease (8 months)
2 (34)	Li ZX	F/10	Right forearm	None	Weakly positive for vimentin; approximately 5% were positive for Ki-67	No genetic testing	Alive with disease (6 months)
3 (35)	El Ochi MR	M/61	Right lung	Surgical resection	diffusely positive staining for vimentin and smooth muscle actin, with focal positivity for CD99.	No genetic testing	Alive with disease (1 months)
4 (36)	Sajko N	M/73	Left thigh	Left thigh	Not described	No genetic testing	Alive with disease (9 months)

Due to their high malignancy, tendency for early metastasis, rapid progression, and poor prognosis, surgery has become the primary treatment method for undifferentiated sarcomas. According to the 8th edition of the AJCC classification for soft tissue sarcomas, this case was classified as stage T1N0M0 II, thus making surgery the preferred treatment (15). Preoperative radiotherapy is an option, with benefits including a lower total dose and reduced irradiation of normal tissue volumes. However, its major drawback is the increased risk of acute wound healing complications. Regardless of the type of resection, excellent local control rates are achieved when preoperative radiotherapy is incorporated into patient care (16, 17).

Treatment options for sarcomas also include chemotherapy. Standard chemotherapy agents for undifferentiated sarcomas include doxorubicin, ifosfamide, gemcitabine, and paclitaxel (18, 19). However, there is currently no standard treatment protocol specifically for USEF, and not all patients with advanced or metastatic soft tissue sarcomas benefit from conventional chemotherapy.

Targeted therapy is pivotal in treating patients resistant to or who have failed conventional chemotherapy. Anlotinib has demonstrated efficacy in refractory metastatic UPS, with a 12-week progression-free rate of 58% and median progression-free survival of 4.1 months (20).

Additionally, in this case, we detected a nonsense mutation p.R1241* in the *NF1* gene, which may lead to NF1 protein inactivation, increasing Ras-GTP levels and activating downstream RAS pathways, thereby promoting excessive cell proliferation. NF1 inactivating mutations might reduce sensitivity to EGFR-targeted drugs, although clinical evidence remains insufficient (21). This mutation could also increase sensitivity to MEK inhibitors such as selumetinib (22).

In this case, the detected p.H214R missense mutation in the *TP53* gene could diminish the tumor-suppressing function of TP53 and is associated with tumor progression and chemotherapy resistance (23). Adavosertib, a WEE1 kinase inhibitor targeting G2 checkpoint control, has shown potential efficacy in preclinical studies against TP53-mutated tumor cells, especially in combination with chemotherapy (24). Further phase II clinical trials have found that Adavosertib significantly enhances chemotherapy sensitivity in patients with TP53 mutations (25). Phase Ib clinical trials have indicated that the combination of Adavosertib and Olaparib is safe and effective in some patients with TP53 mutations (26). These findings underscore the importance of TP53 mutations in guiding drug choices for this case, with Adavosertib being a potential treatment option.

The NCCN Clinical Practice Guidelines recommend pembrolizumab, an immune checkpoint inhibitor, for the treatment of undifferentiated sarcomas (27). The high TMB detected in this case further enhances the potential for immunotherapy. Exome sequencing revealed a TMB of 16.5 Muts/Mb, suggesting that the patient may respond well to pembrolizumab immunotherapy. FDA has approved pembrolizumab for the treatment of patients with solid tumors with high TMB (TMB-H) (28). In the KEYNOTE-158 trial, patients with high TMB showed significant overall response rates (29). TP53 mutations might be positive predictors for immunotherapy, as such genetic alterations can lead to increased PD-L1 expression (30). Patients with TP53 mutations, especially those with concurrent TP53/KRAS mutations, could significantly benefit from PD-1 inhibitors (31, 32). Therefore, considering the high TMB and presence of TP53 mutation, pembrolizumab immunotherapy emerges as a promising treatment option for this case. We summarize the treatment strategies for unclassified sarcomas with epithelioid features outlining treatment in Table 3.

**Table 3:** Overview of treatment strategies for unclassified sarcoma with epithelioid features therapy.

Treatment strategies		Descriptions
Surgery		Surgery is the primary treatment method for undifferentiated sarcomas (15).
Preoperative radiotherapy		Preoperative radiotherapy, offering benefits such as lower dosage and minimal irradiation of normal tissues, is an effective option. Excellent local control rates are consistently achieved with this approach, regardless of the resection type (16, 17).
Chemotherapy		Standard chemotherapy agents for undifferentiated sarcomas include doxorubicin, ifosfamide, gemcitabine, and paclitaxel (18, 19).
Targeted therapy	Erlotinib and gefitinib	NF1 inactivating mutations might reduce sensitivity to EGFR-targeted drugs, although clinical evidence is not yet sufficient (21)
	Selumetinib	NF1 inactivating mutations increase sensitivity to MEK inhibitors like Selumetinib (22)
	Adavosertib	Adavosertib, a WEE1 kinase inhibitor, has demonstrated effectiveness in enhancing chemotherapy sensitivity in TP53-mutated tumor cells, suggesting its potential as a treatment option for tumors with TP53 mutations (24).
Immunotherapy	Pembrolizumab	Pembrolizumab has been approved by the FDA for treating patients with high Tumor Mutation Burden (TMB-H) solid tumors, as these patients have shown significant overall response rates in the KEYNOTE-158 trial (28, 29).
		TP53 mutations, which can lead to increased PD-L1 expression, may serve as positive predictors for the effectiveness of pembrolizumab immunotherapy, especially in patients with concurrent TP53/KRAS mutations (30–32).

Tumor DNA sequencing serves a dual purpose: guiding drug selection and providing prognostic insights into the disease course. In this case, we detected a *TP53* mutation in the patient. This mutation, identified as pathogenic in multiple databases, including UMD, is anticipated to reduce the tumor-suppressive function of the *TP53* gene. It is likely involved in tumor development, progression, and poor prognosis. To discuss the impact of *TP53* mutations on tumor progression, especially given the rapid progression of the tumor in this case, in Table 4, we incorporate *TP53* mutations that show a positive correlation with outcomes or overall survival.

**Table 4:** TP53 mutation types and their impact on survival and prognosis across three different types of cancers.

Cancer type	TP53 mutation type	Survival impact	Prognostic impact	Notes
Diffuse large B-cell lymphoma (DLBCL)	74% missense mutations, predominantly in exons 5–8 DNA-binding domain. High-frequency mutations at codons 175, 273, 248	No significant impact on overall survival (OS), poorer prognosis in GCB subtype	TP53WT&CD58MUT with worst OS (median 92.3 months); TP53MUT&CD58WT with best OS (median 110.8 months)	TP53 mutation rate: 30% (37)
Hepatocellular carcinoma (HCC)	63.3% missense, 20.2% nonsense, 16.5% frameshift mutations. Hotspots at R249S, R175H, R273H, R248Q, R282W	High-risk group with median survival 2.46 years, low-risk group 6.81 years	TP53MLncSig prognostic model differentiates high and low-risk patients, correlates with response to immune checkpoint blockade (ICB) therapy	TP53 mutation rate: 29% (38)
Breast cancer	Inactive (64.7%), Structural (23.5%, e.g., R175H, G245S, R249S, Y220C), Contact (11.8%, e.g., R273H/C, R248W)	Inactive: OS median 12.5 months, PFS 5.5 months; Structural: OS median 22.5 months, PFS 10.5 months; Contact: OS median 24.5 months, PFS 11.5 months	Inactive type associated with significantly worse prognosis compared to Contact and Structural types	(39)

This case represents the first instance of USEF originating from the pleura encountered by our team, marked by its rapid development and challenges in detection. Despite the patient’s decision to forego antineoplastic treatment, the severity and invasiveness of USEF were underscored by the disease’s swift advancement, culminating in the patient’s death due to respiratory failure. Given that the patient did not receive any form of treatment, this report primarily focuses on the diagnosis of USEF and the discussions surrounding potential therapeutic implications based on the patient’s NGS results. In this context, we thoroughly analyze the clinical and pathological characteristics of this specific case, contemplate the patient’s potential treatment options, and provide insights for future care strategies in similar scenarios.

## Data availability statement

The datasets presented in this study can be found in online repositories. The names of the repository/repositories and accession number(s) can be found at: https://www.ncbi.nlm.nih.gov/genbank/, PRJNA1063166.

## Ethics statement

The studies involving humans were approved by Institutional Review Board (IRB) of the Second Affiliated Hospital of Chongqing Medical University. The studies were conducted in accordance with the local legislation and institutional requirements. The participants provided their written informed consent to participate in this study. Written informed consent was obtained from the individual(s) for the publication of any potentially identifiable images or data included in this article.

## Author contributions

L-XX: Conceptualization, Data curation, Visualization, Writing – original draft. LL: Formal analysis, Supervision, Writing – review & editing. WD: Funding acquisition, Investigation, Project administration, Supervision, Writing – review & editing.
