# Supplementary material for: Case report: The first account of undifferentiated sarcoma with epithelioid features originating in the pleura
Source: Front Med (Lausanne). 2024 Feb 1;11:1301941. doi: 10.3389/fmed.2024.1301941 (PMC10867128; doi:10.3389/fmed.2024.1301941)
Supplement: Supplementary file 2 [file Data_Sheet_1.zip › Data Sheet 1.docx]

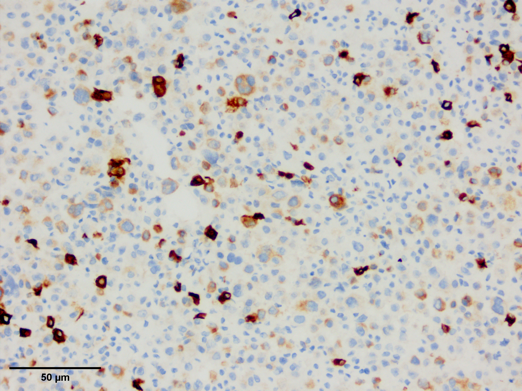

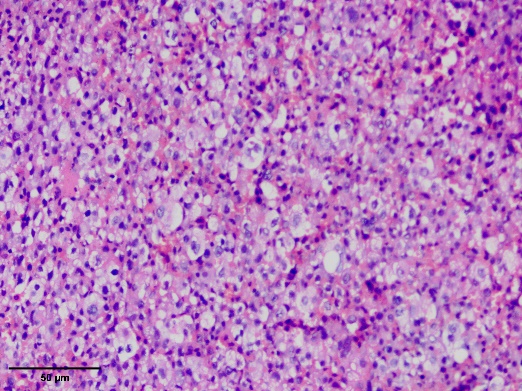


A B


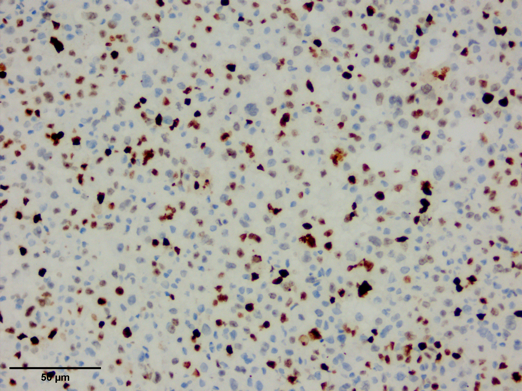

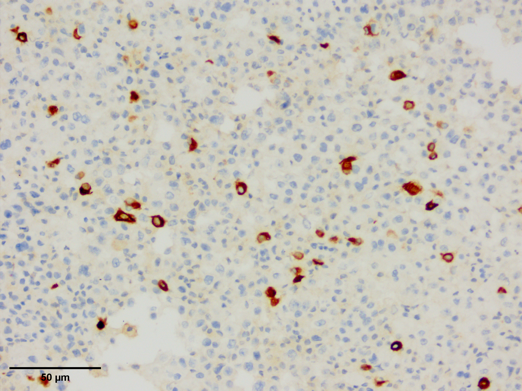


C D


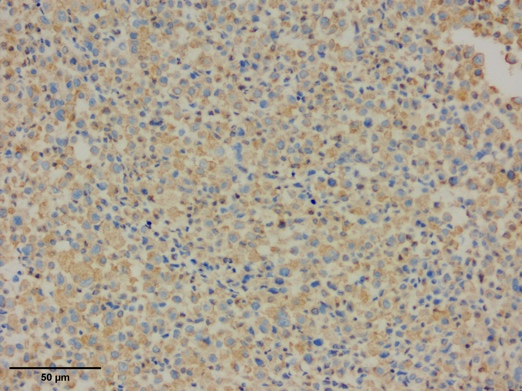

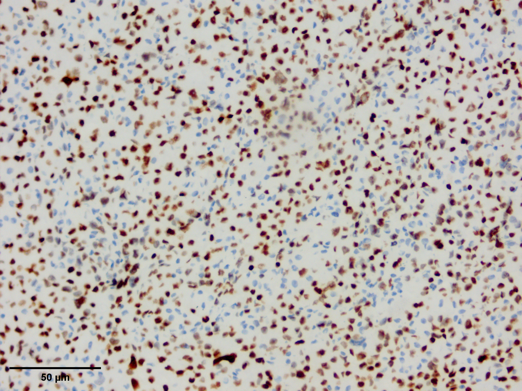


E F

Figure S1 (A): Hematoxylin and Eosin (HE) staining at 20x magnification illustrates the features of the paraffin-embedded specimen obtained from the right pleural effusion. (B): Immunohistochemical staining for Cytokeratin (CK) demonstrates positive staining in small foci.(C): Immunohistochemical staining for CK7 showing focal positive staining.(D): Immunohistochemical staining for Ki-67 indicates a proliferation index with 30% positivity.(E): Immunohistochemical staining for Thyroid Transcription Factor-1 (TTF-1) exhibits positive results.(F): Immunohistochemical staining for Vimentin (Vim) demonstrates positive staining.


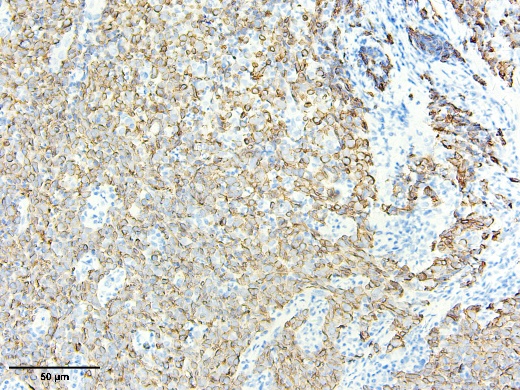

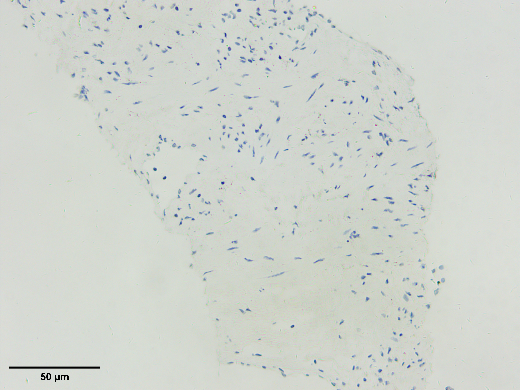


A B


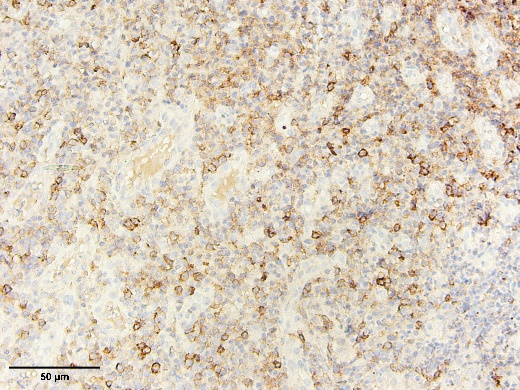

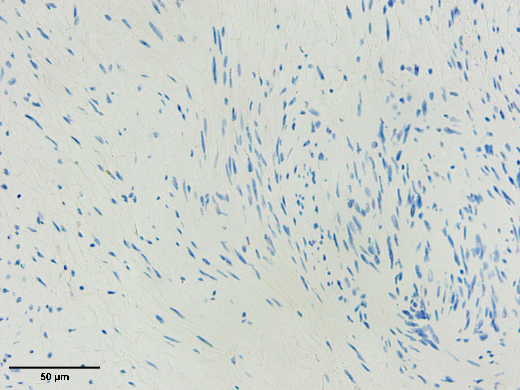


C D


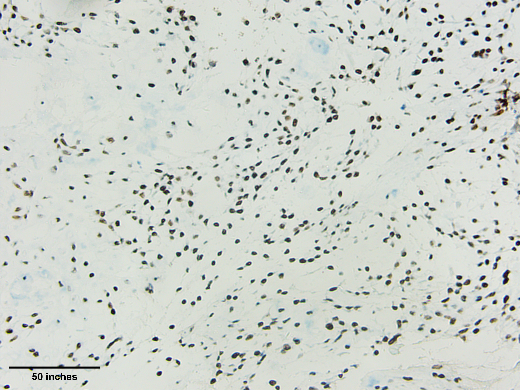

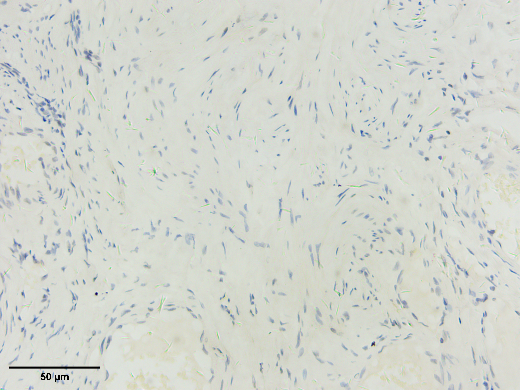


E F


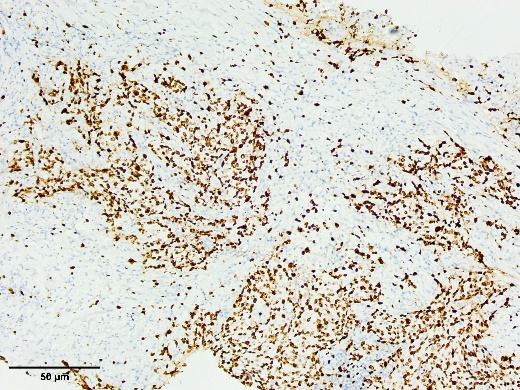

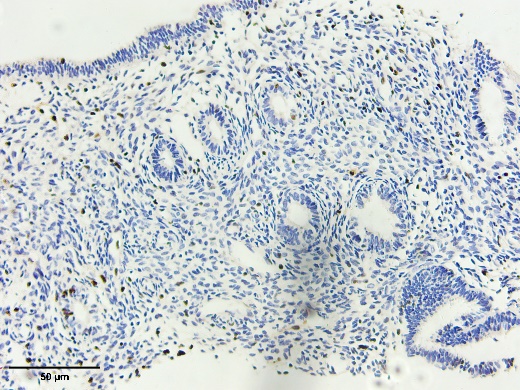


G H


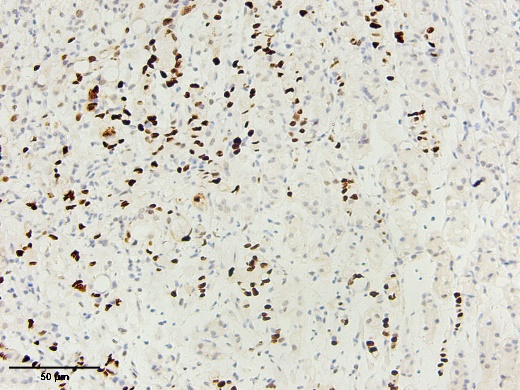

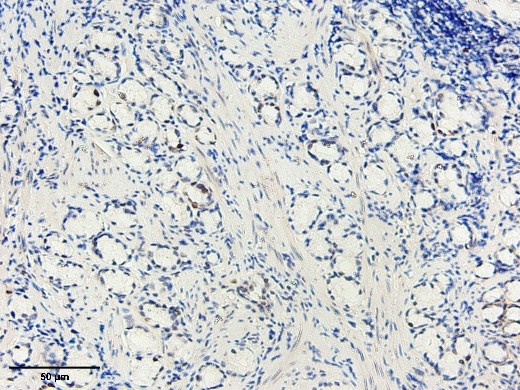


I J


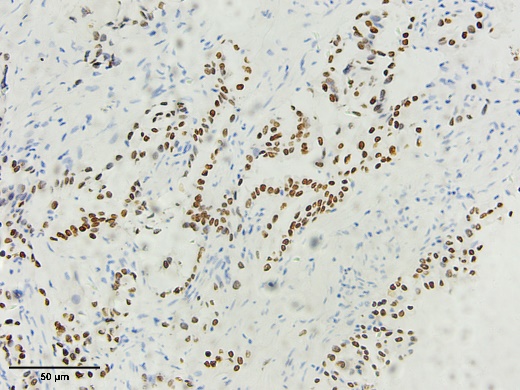

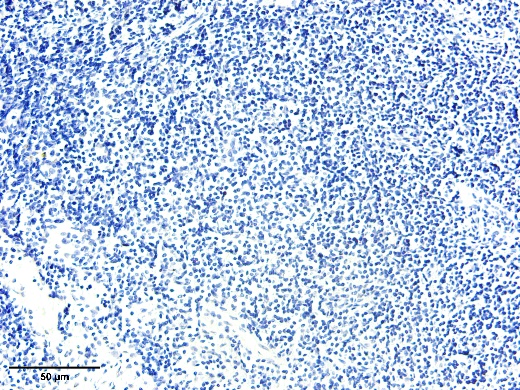


K L


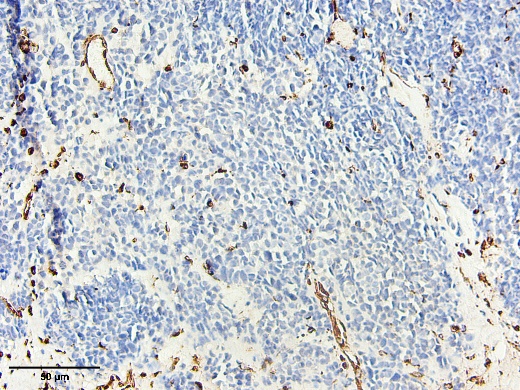

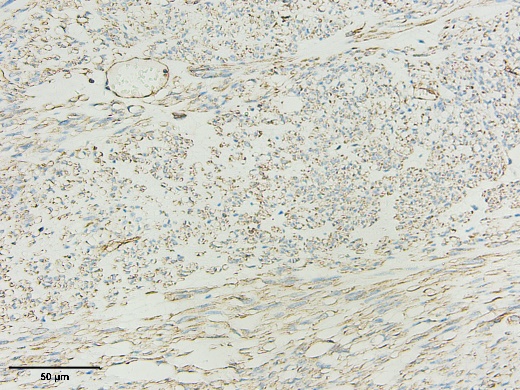


M N

Figure S2: Immunohistochemical Staining Demonstrating Positive and Negative Controls for Specific Markers

(A): Immunohistochemical staining for CK5/6 showing positive staining in nasopharyngeal squamous carcinoma.(B): Immunohistochemical staining for CK5/6 demonstrates negative control in prostate acinar adenocarcinoma.(C): Immunohistochemical staining for EMA indicates positive staining in esophageal adjacent tissue.(D): Immunohistochemical staining for EMA shows negative control in lower leg schwannoma.(E): Immunohistochemical staining for INI-1 presents positive results in osteosarcoma.(F): Immunohistochemical staining for INI-1 as a negative control in hemangioma.(G): Immunohistochemical staining for Ki-67 illustrating high proliferation index in breast cancer.(H): Immunohistochemical staining for Ki-67 as a negative control in endometrium.(I): Immunohistochemical staining for P53 exhibiting positive staining in gastric body adenocarcinoma.(J): Immunohistochemical staining for P53 as a negative control in gastric antrum.(K): Immunohistochemical staining for TTF-1 showing positive staining in pleural metastatic adenocarcinoma.(L): Immunohistochemical staining for TTF-1 as a negative control in pre-laryngeal lymph node.(M): Immunohistochemical staining for vimentin indicating positive results in gastrointestinal stromal tumor.(N): Immunohistochemical staining for vimentin as a negative control in small cell lung cancer.
