# Supplementary figures and images for: Case report: The first account of undifferentiated sarcoma with epithelioid features originating in the pleura
Source: Front Med (Lausanne). 2024 Feb 1;11:1301941. doi: 10.3389/fmed.2024.1301941 (PMC10867128; doi:10.3389/fmed.2024.1301941)

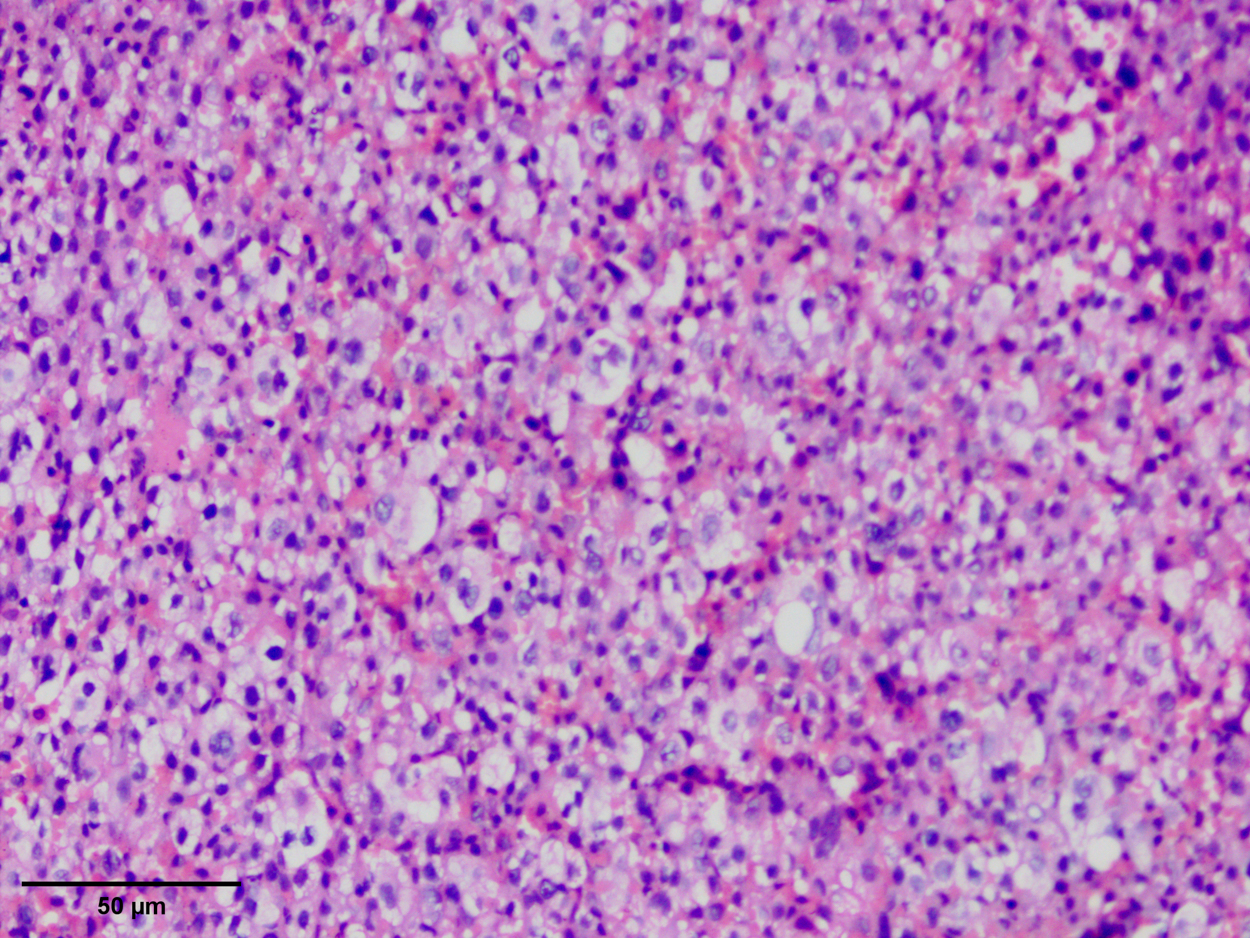

Supplement: Supplementary file 2 [file Data_Sheet_1.zip › 300dpi/Figure S1 (A).JPG]

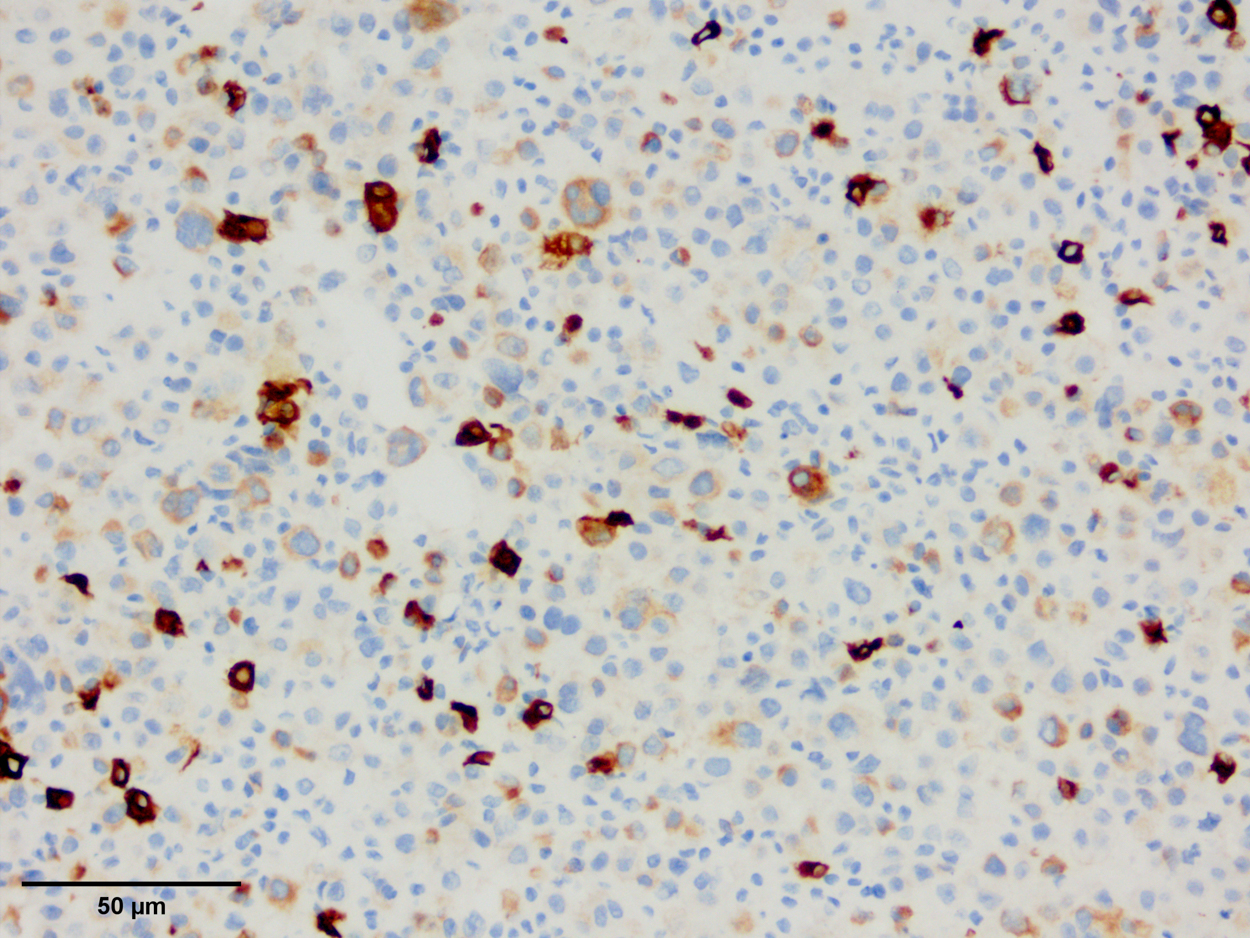

Supplement: Supplementary file 2 [file Data_Sheet_1.zip › 300dpi/Figure S1 (B).JPG]

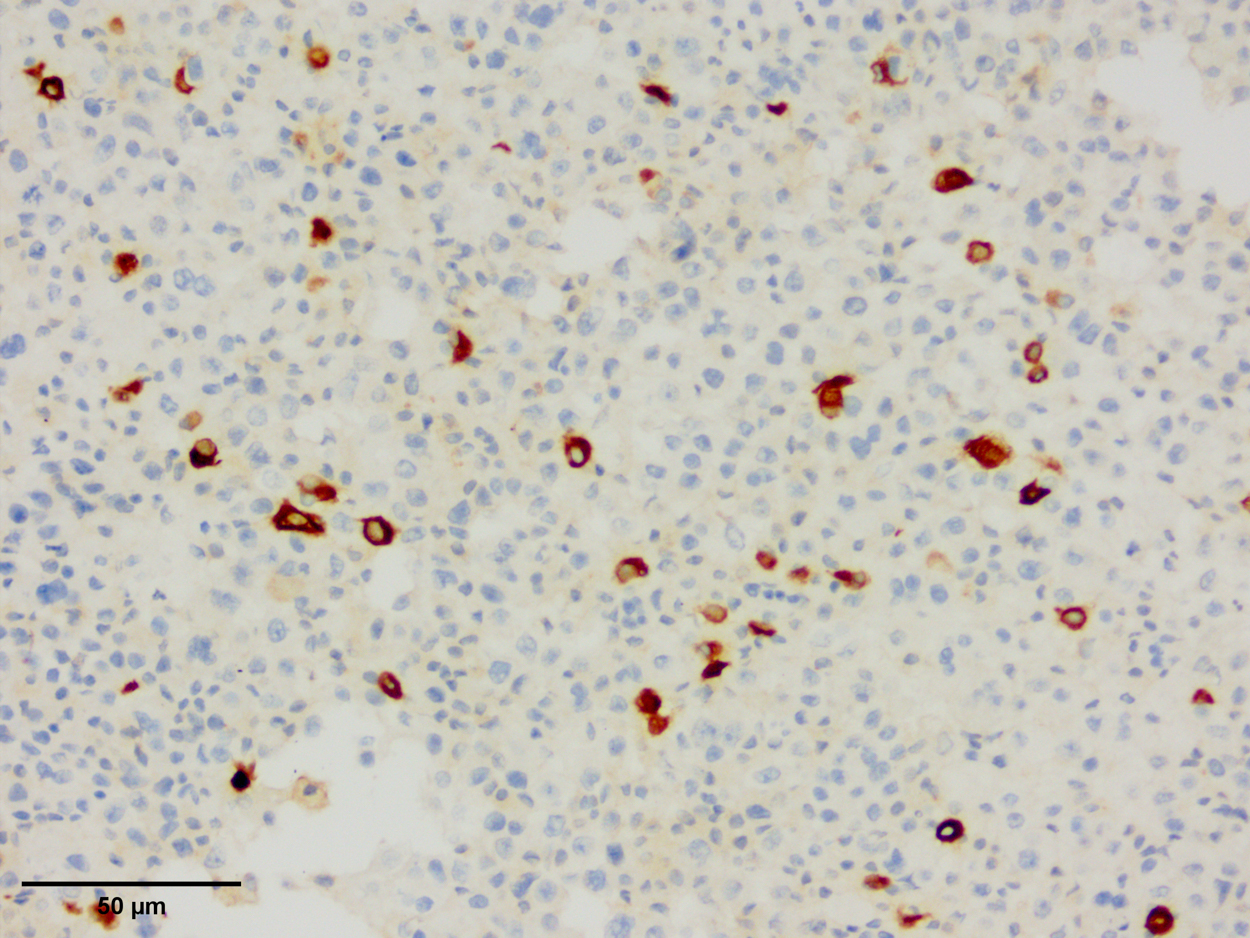

Supplement: Supplementary file 2 [file Data_Sheet_1.zip › 300dpi/Figure S1 (C).JPG]

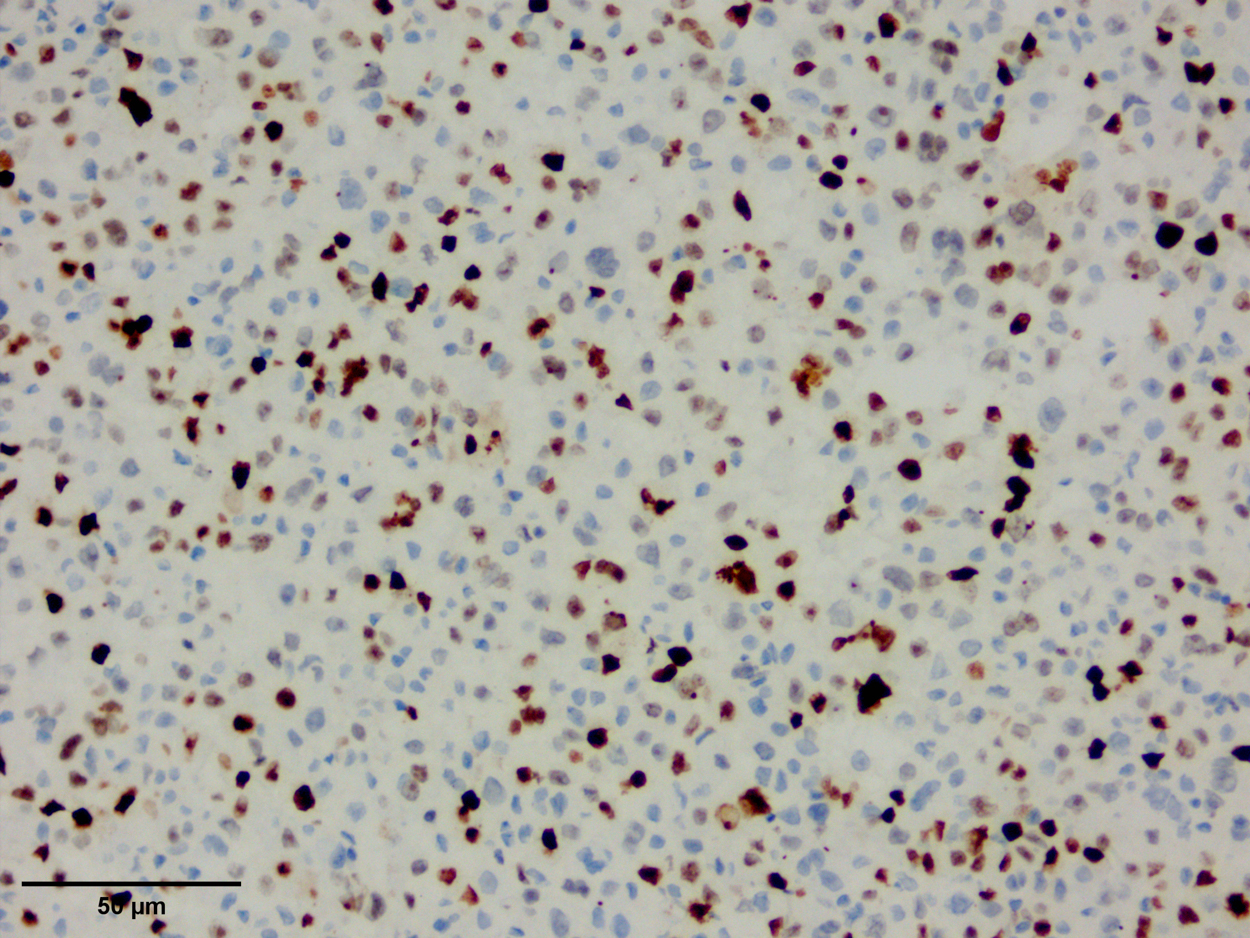

Supplement: Supplementary file 2 [file Data_Sheet_1.zip › 300dpi/Figure S1 (D).JPG]

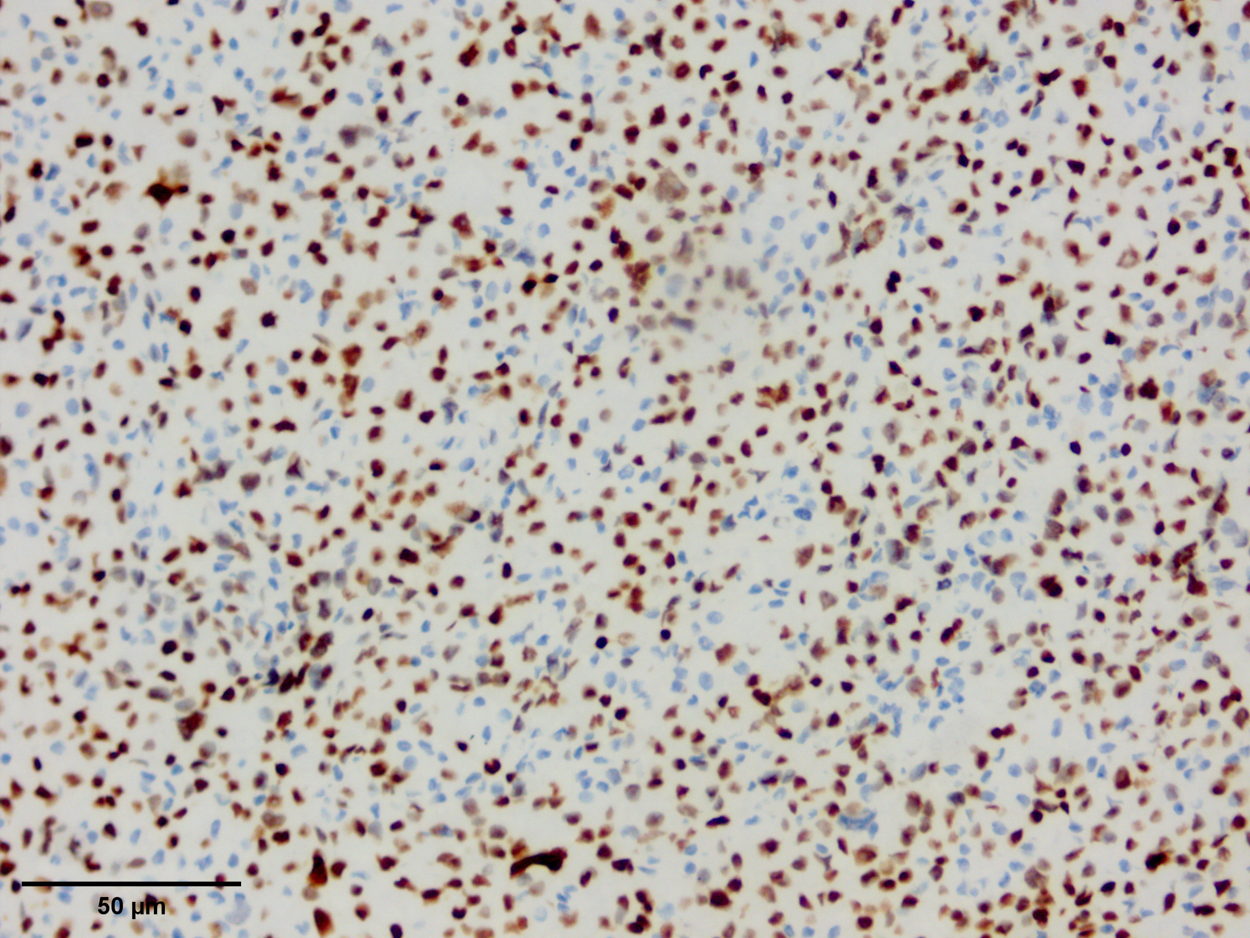

Supplement: Supplementary file 2 [file Data_Sheet_1.zip › 300dpi/Figure S1 (E).JPG]

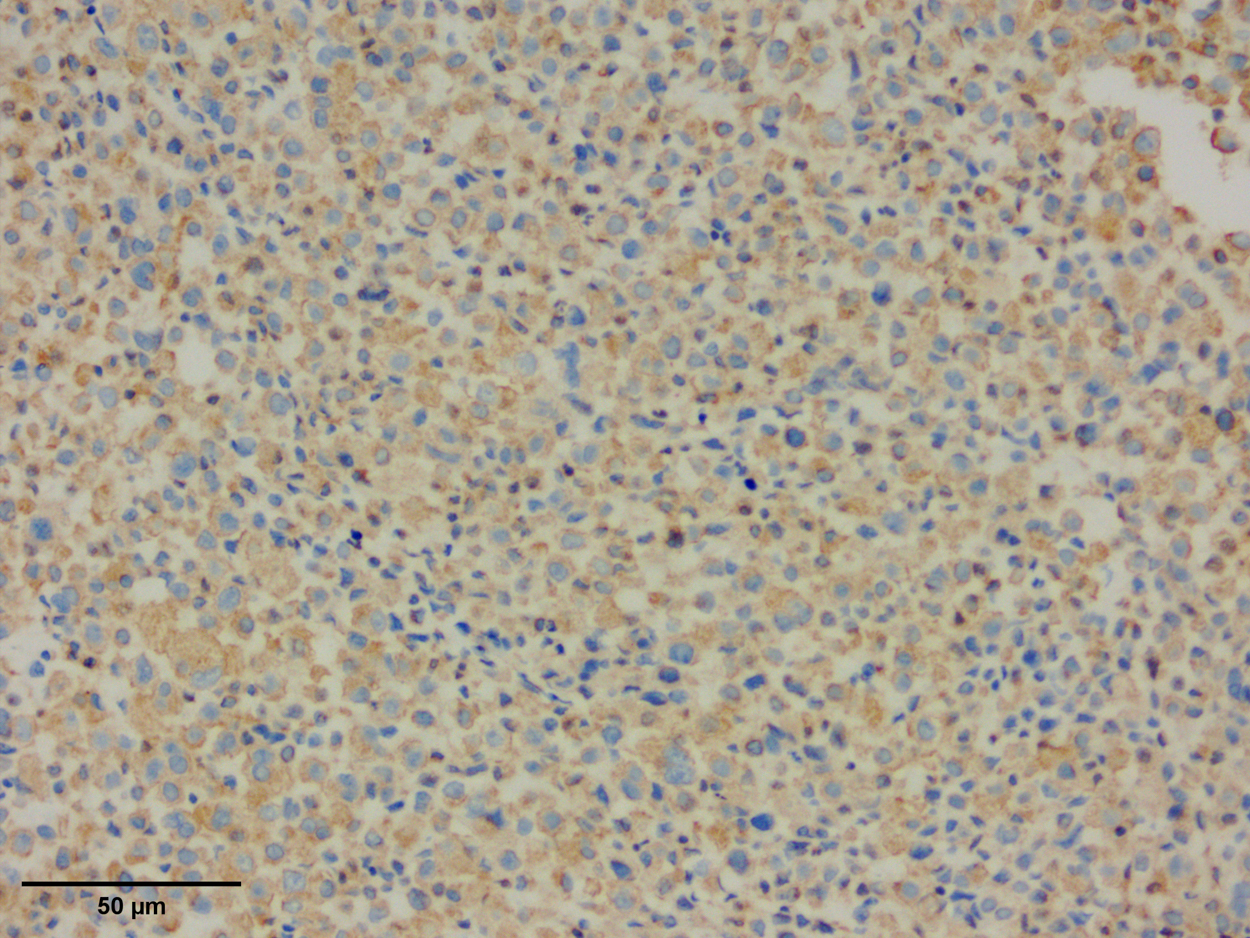

Supplement: Supplementary file 2 [file Data_Sheet_1.zip › 300dpi/Figure S1 (F).JPG]

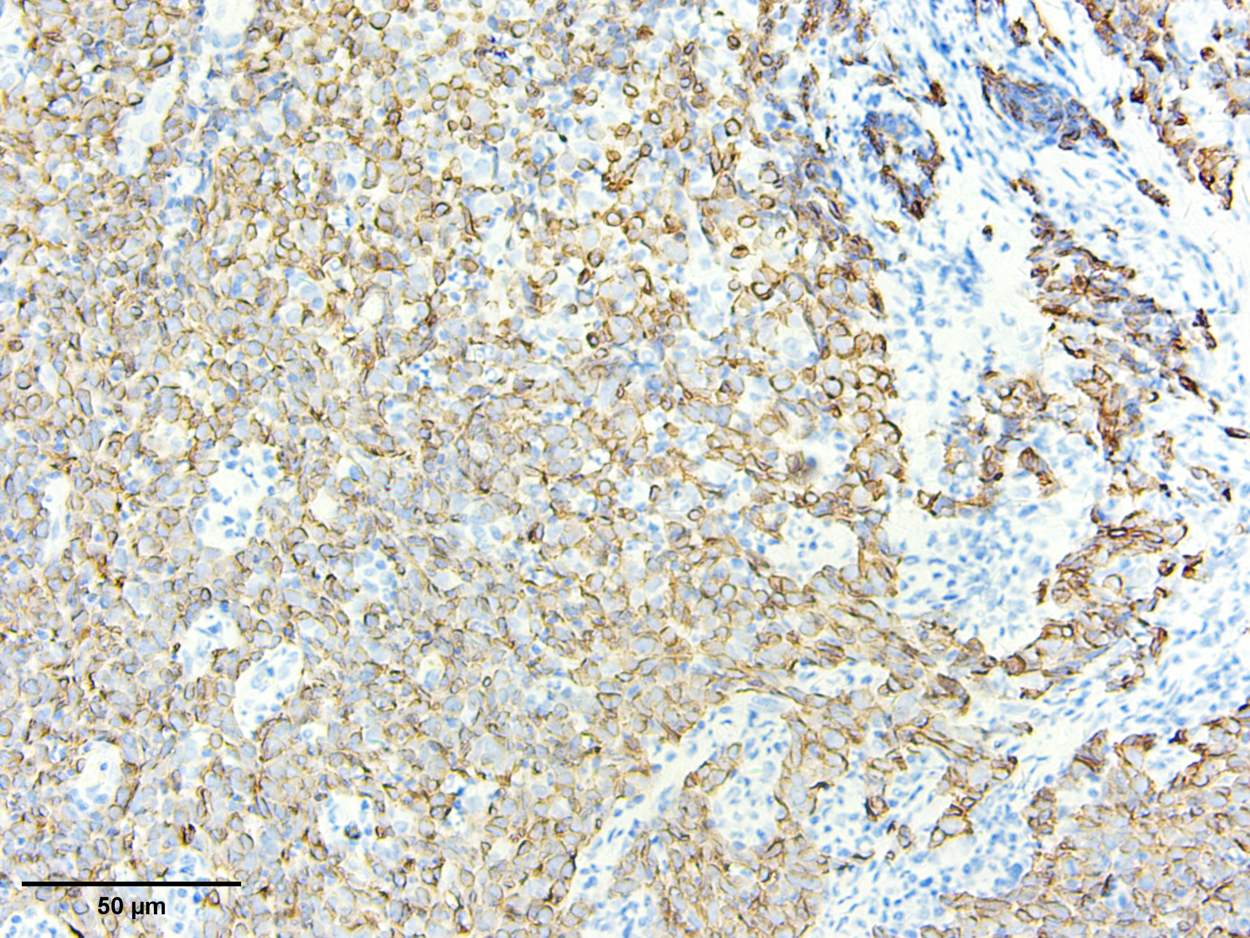

Supplement: Supplementary file 2 [file Data_Sheet_1.zip › 300dpi/Figure S2 A.JPG]

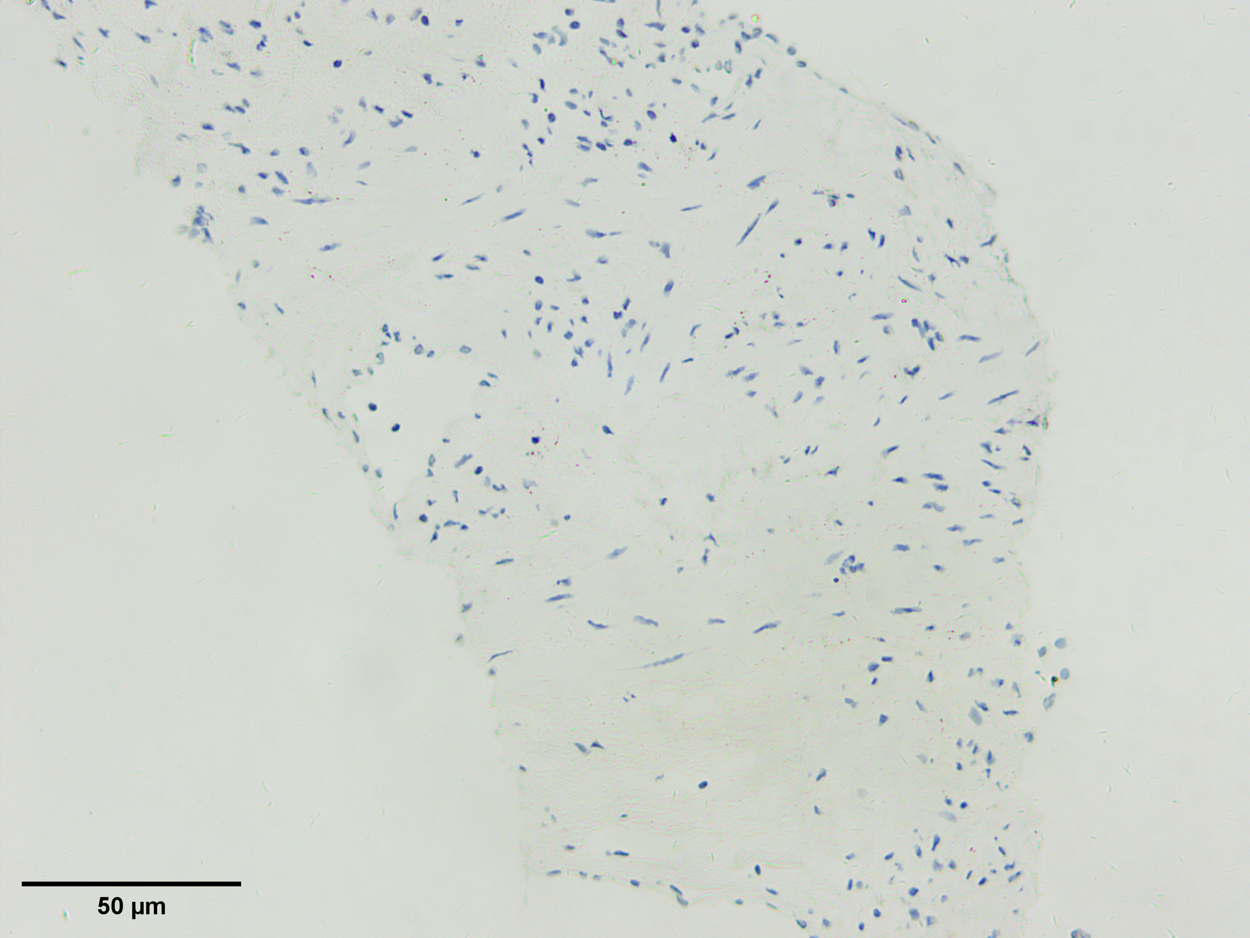

Supplement: Supplementary file 2 [file Data_Sheet_1.zip › 300dpi/Figure S2 B.JPG]

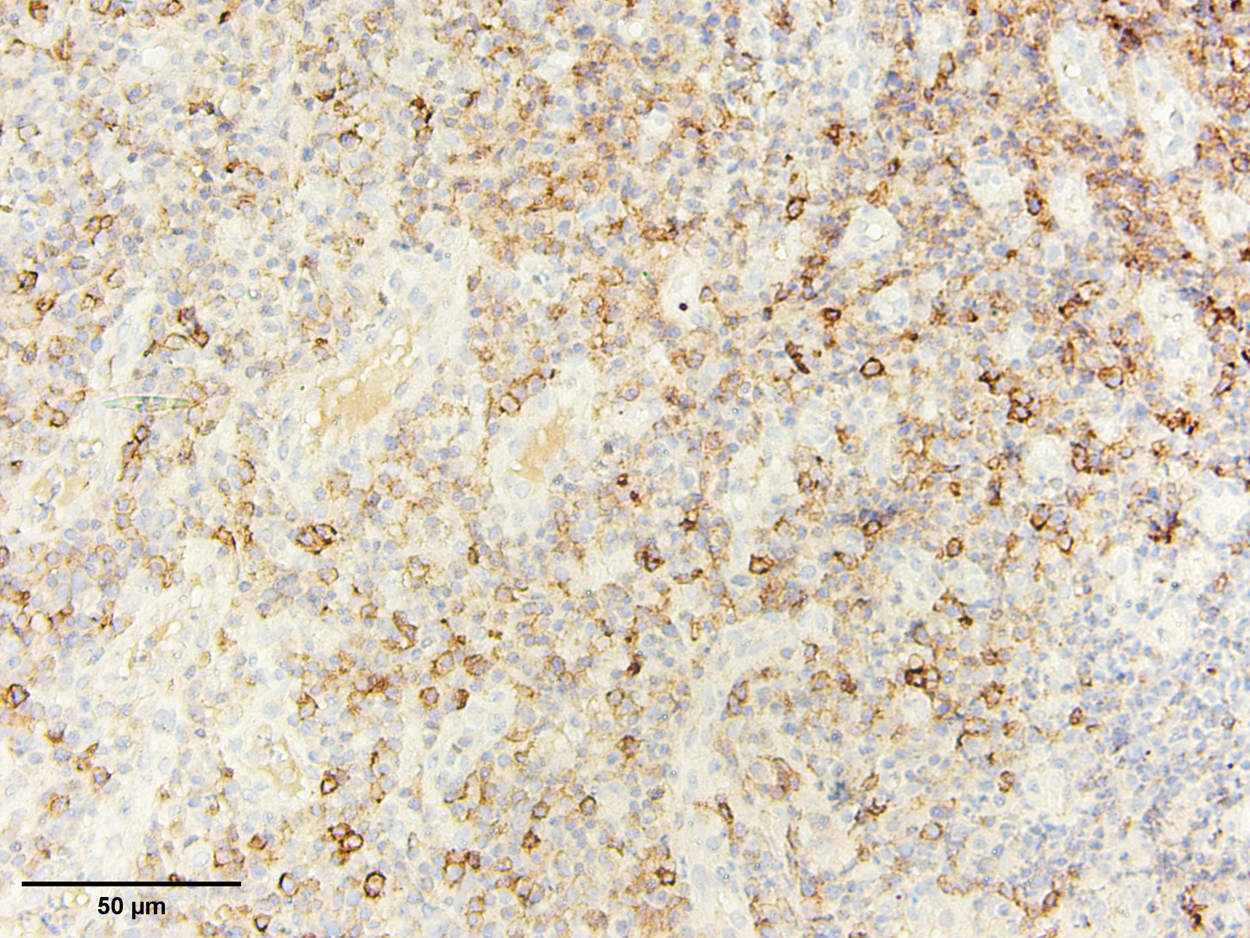

Supplement: Supplementary file 2 [file Data_Sheet_1.zip › 300dpi/Figure S2 C.JPG]

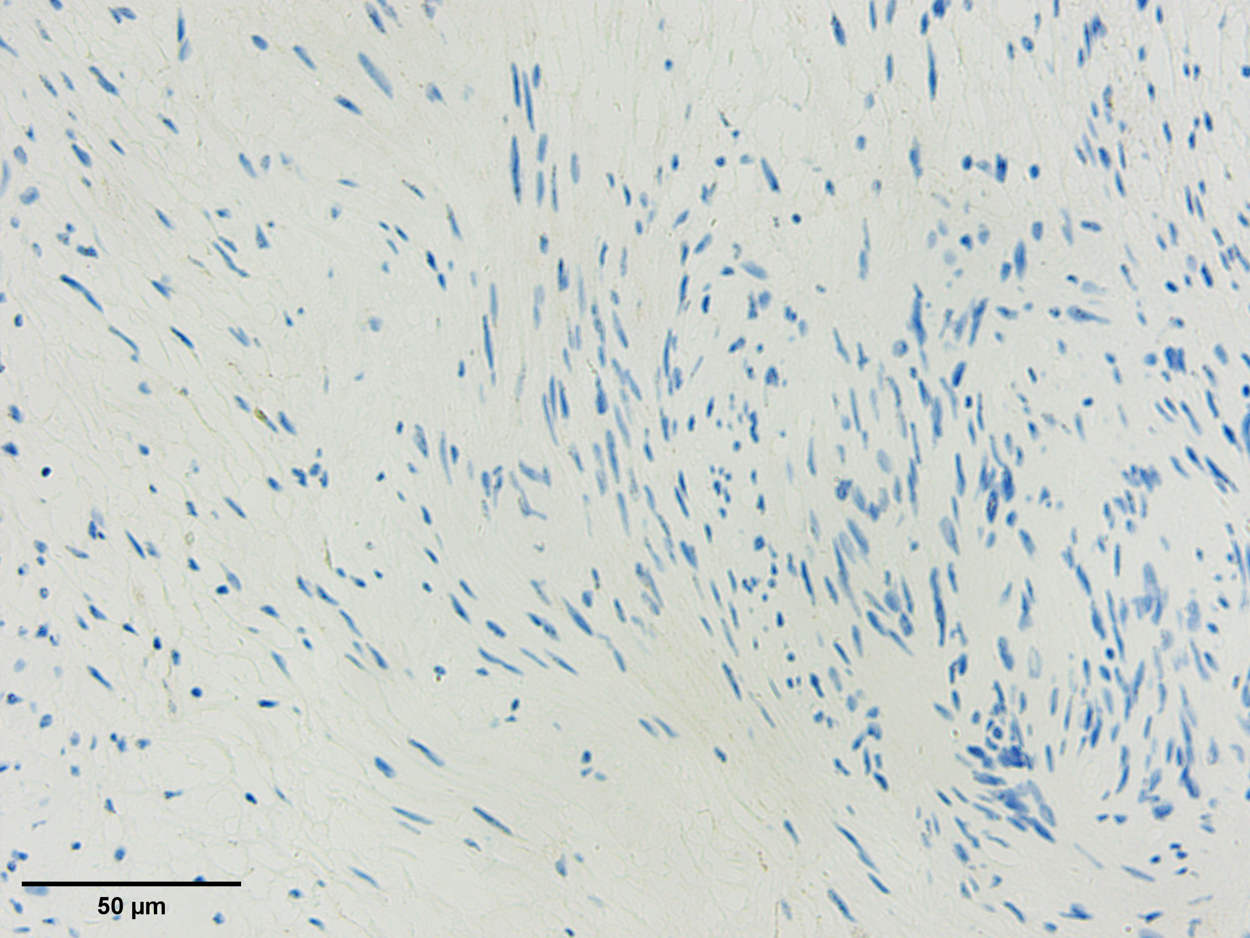

Supplement: Supplementary file 2 [file Data_Sheet_1.zip › 300dpi/Figure S2 D.JPG]

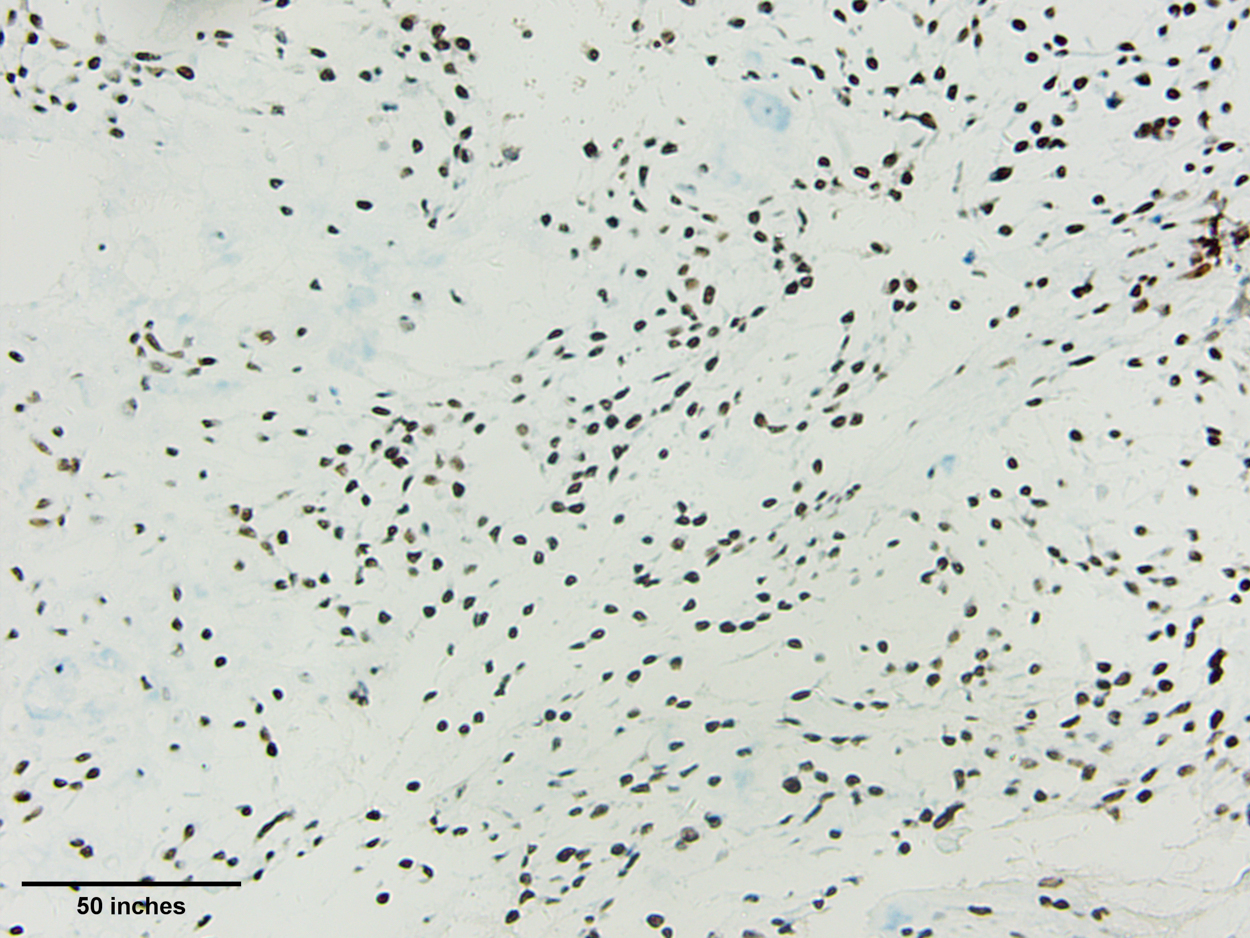

Supplement: Supplementary file 2 [file Data_Sheet_1.zip › 300dpi/Figure S2 E.JPG]

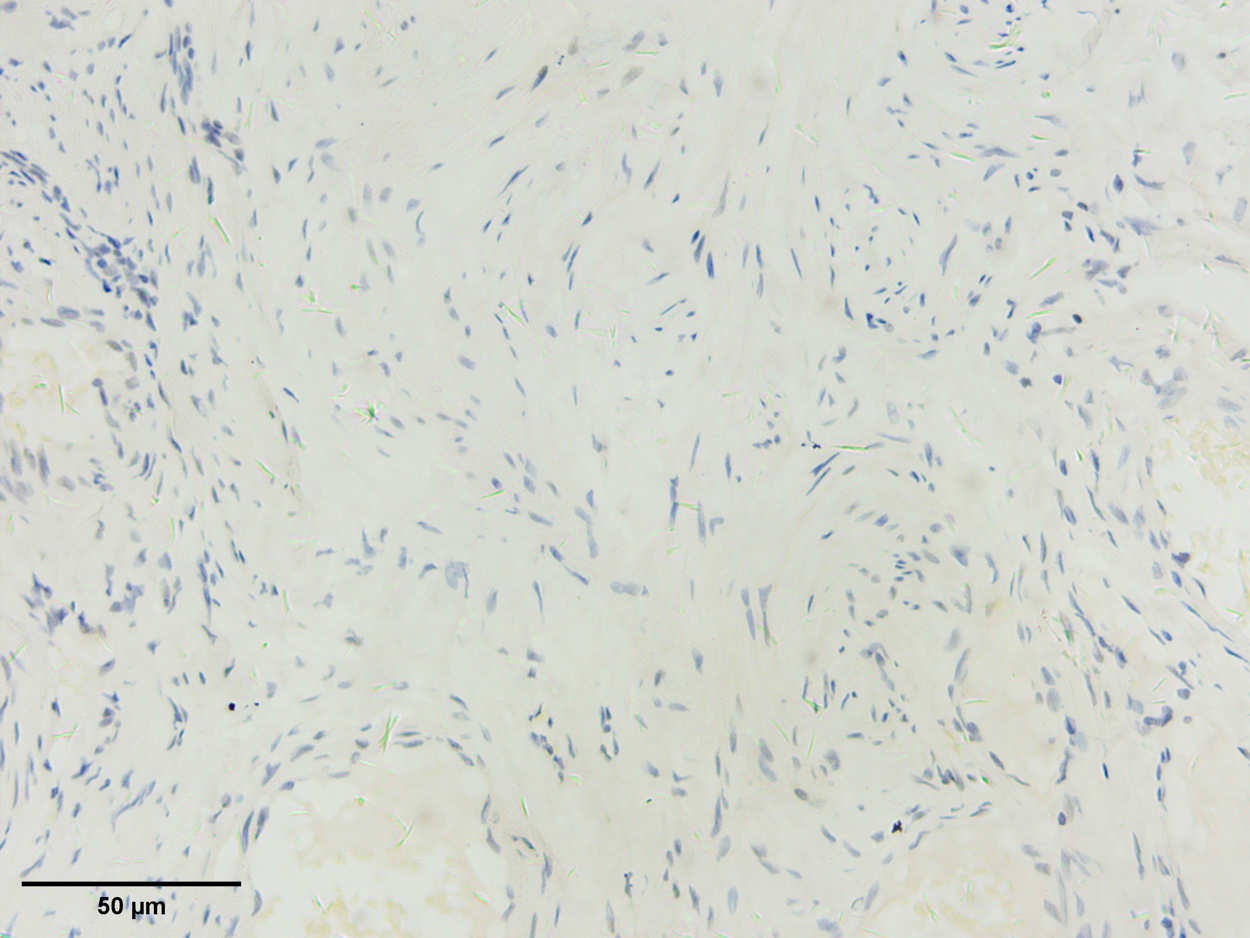

Supplement: Supplementary file 2 [file Data_Sheet_1.zip › 300dpi/Figure S2 F.JPG]

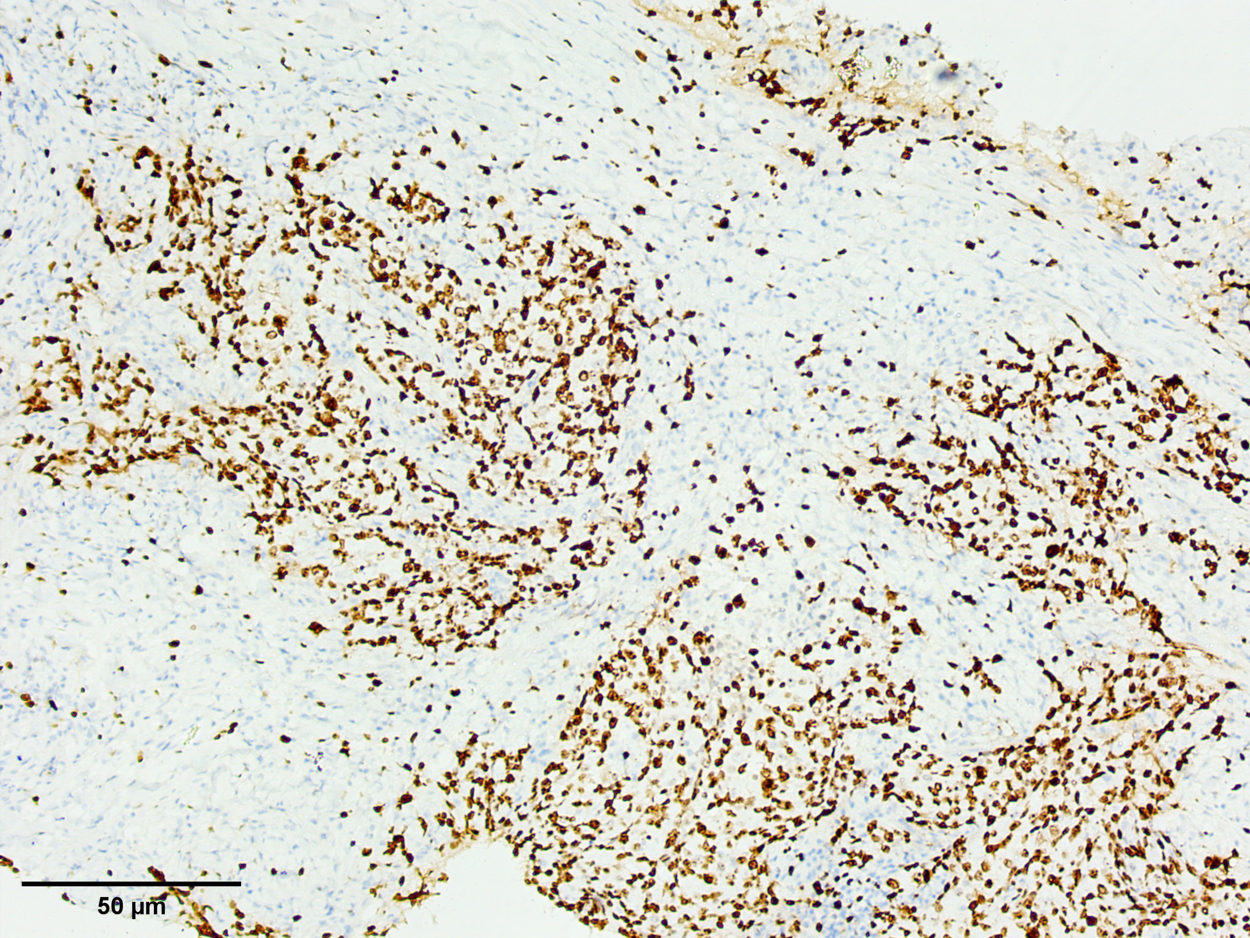

Supplement: Supplementary file 2 [file Data_Sheet_1.zip › 300dpi/Figure S2 G.JPG]

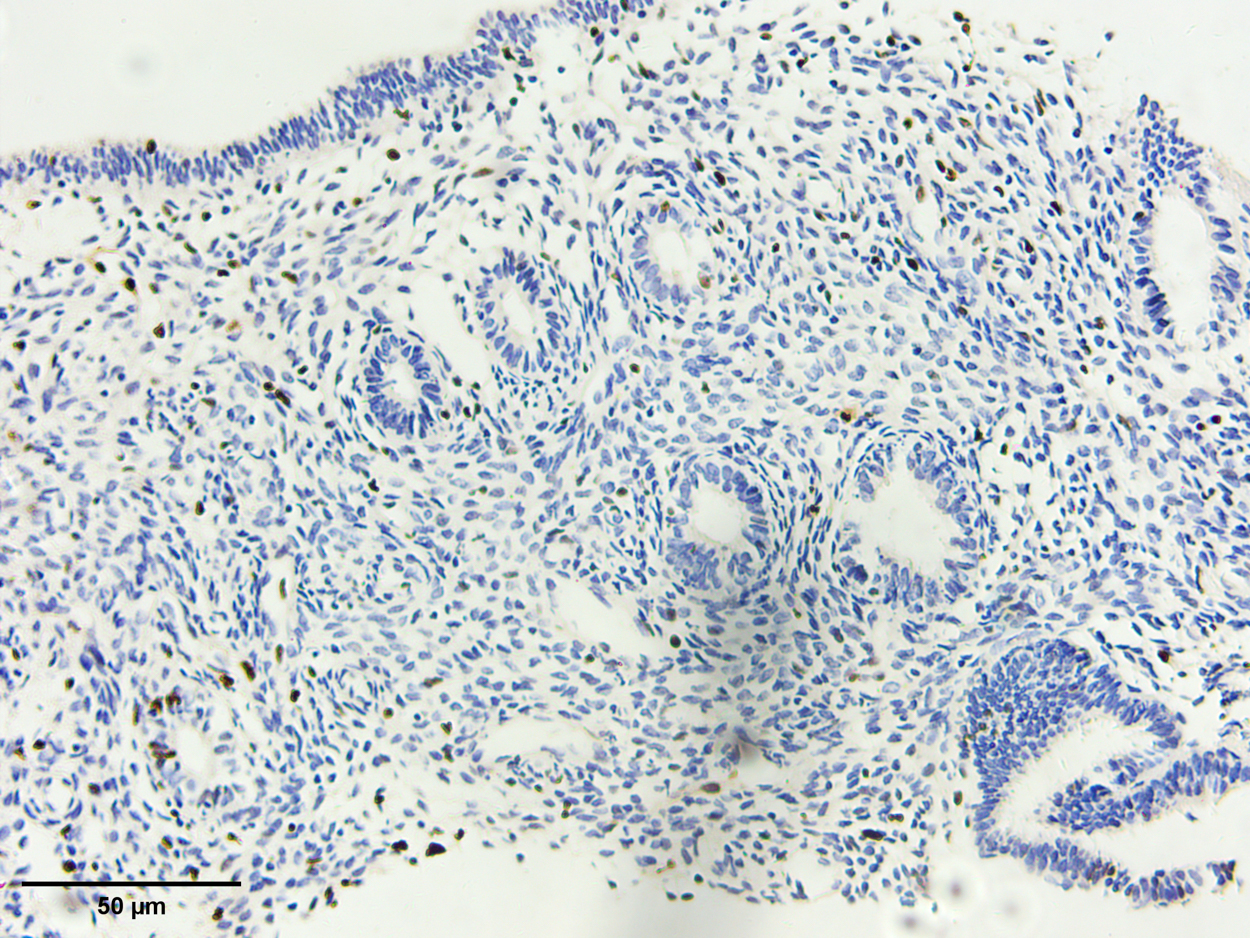

Supplement: Supplementary file 2 [file Data_Sheet_1.zip › 300dpi/Figure S2 H.JPG]

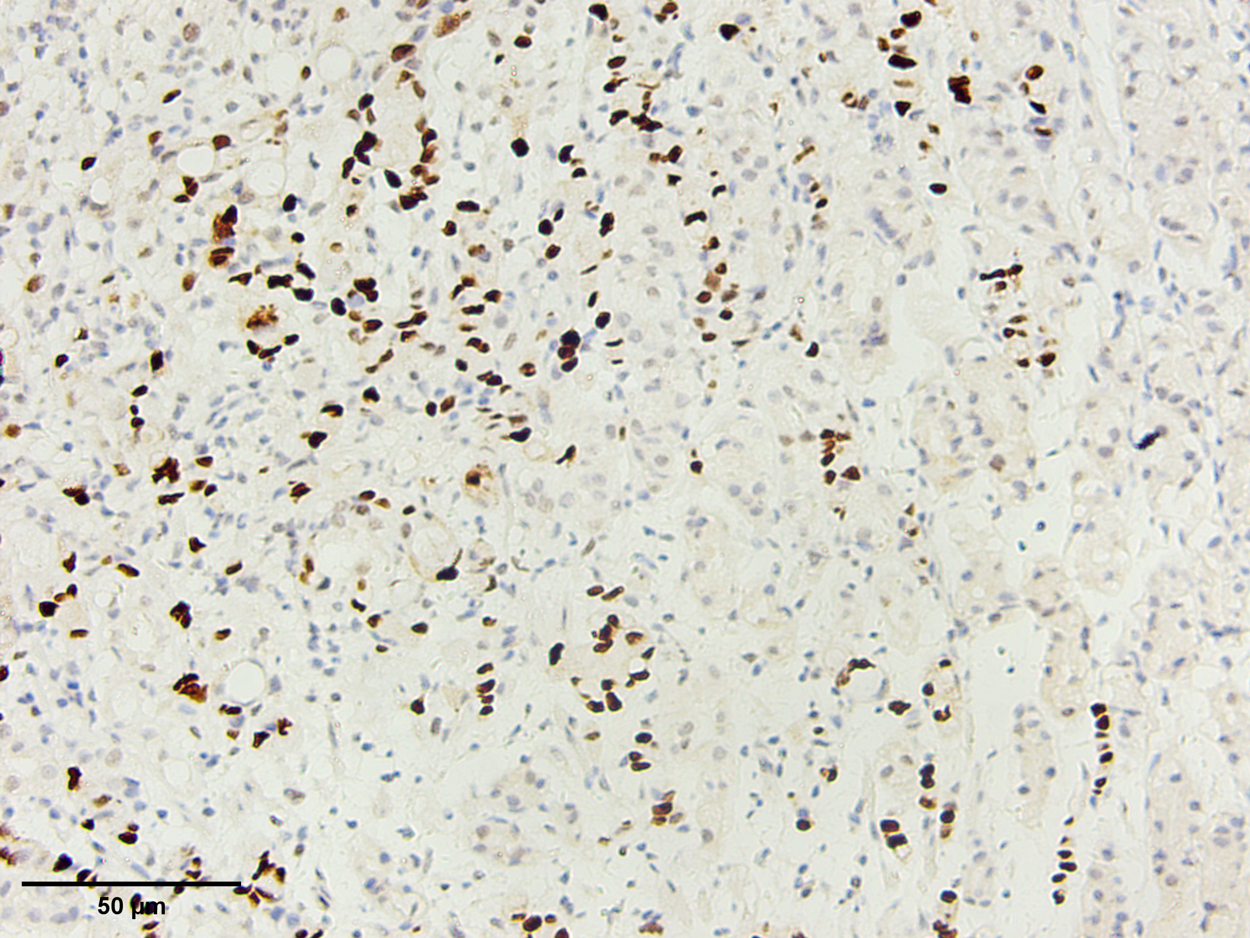

Supplement: Supplementary file 2 [file Data_Sheet_1.zip › 300dpi/Figure S2 I.JPG]

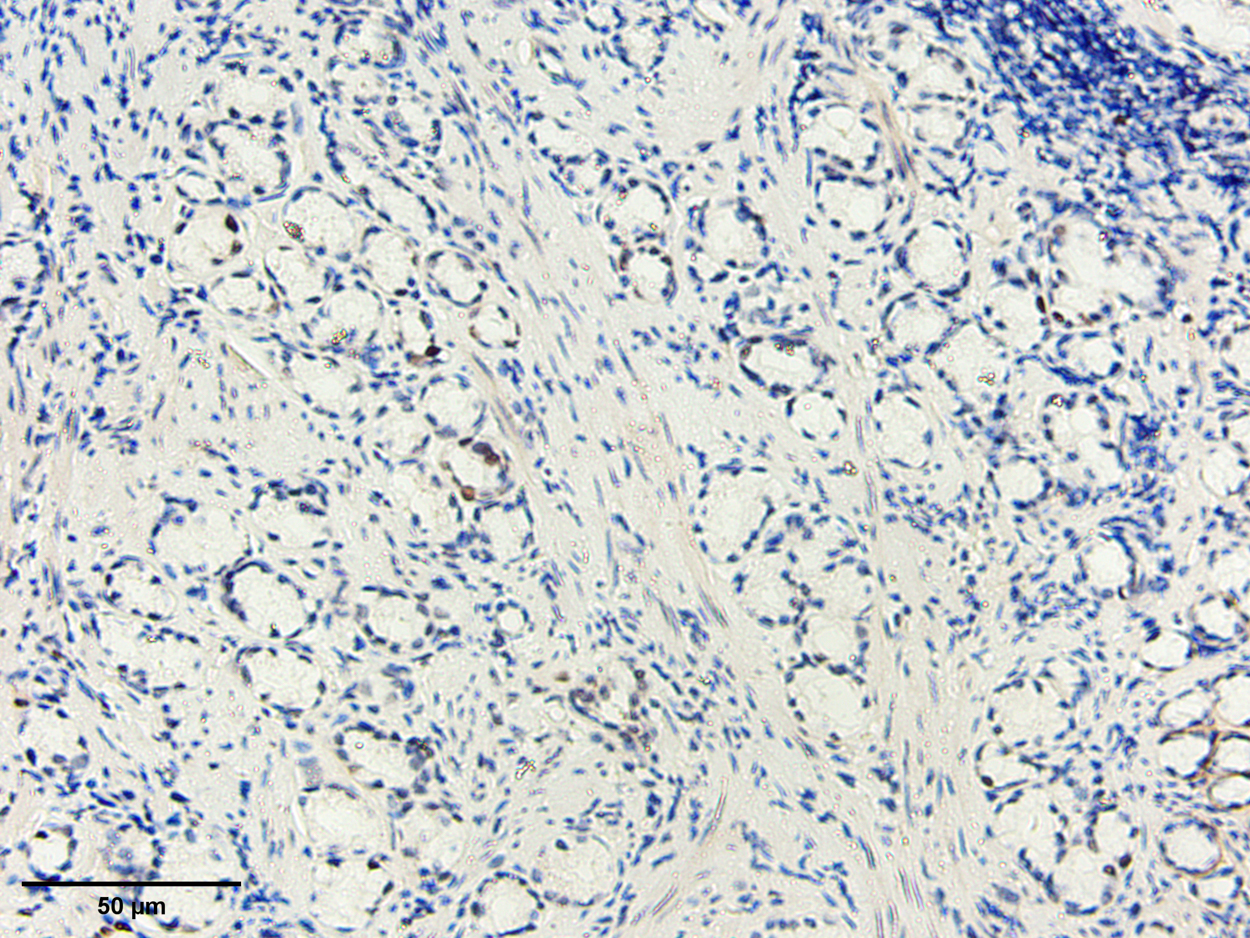

Supplement: Supplementary file 2 [file Data_Sheet_1.zip › 300dpi/Figure S2 J.JPG]

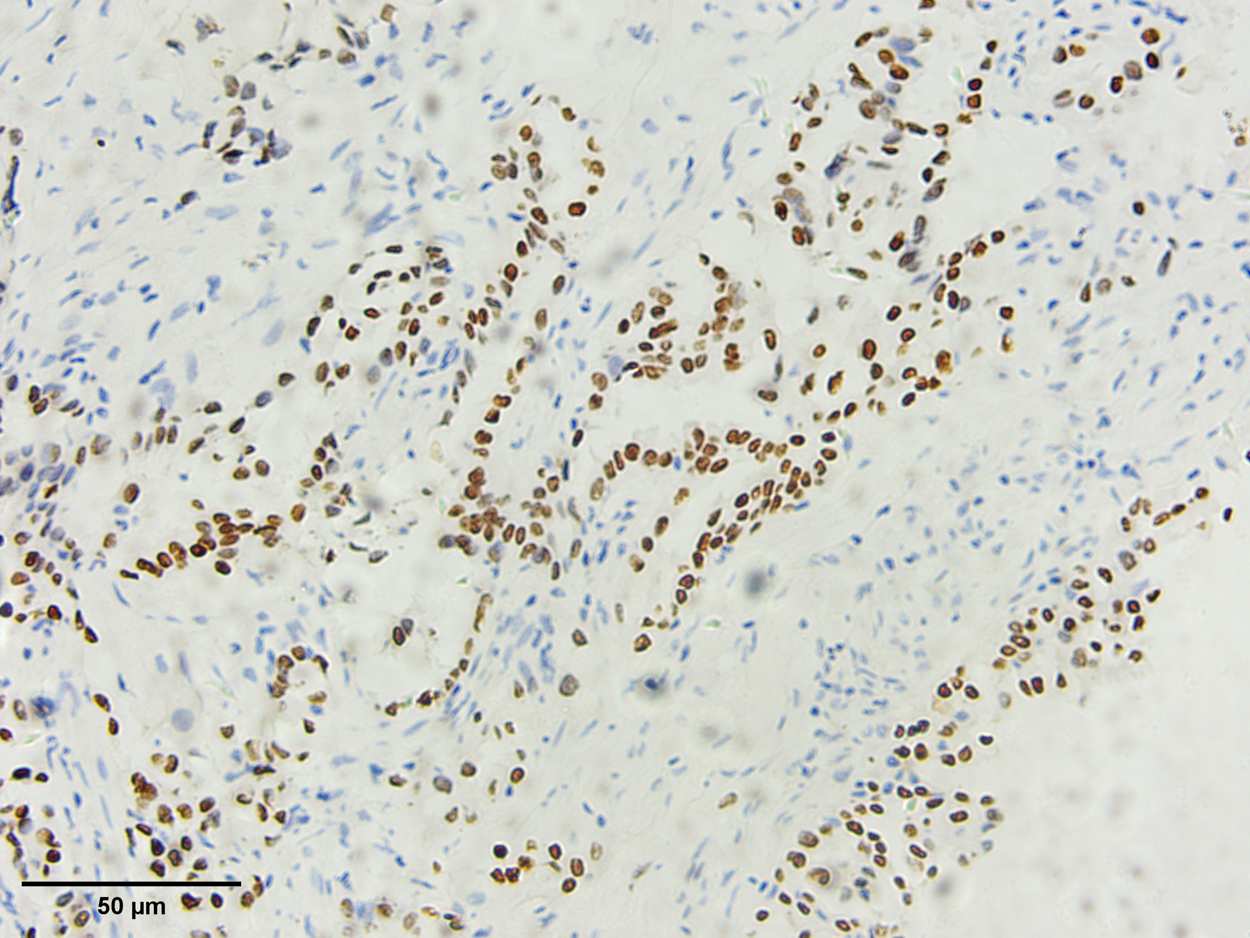

Supplement: Supplementary file 2 [file Data_Sheet_1.zip › 300dpi/Figure S2 K.JPG]

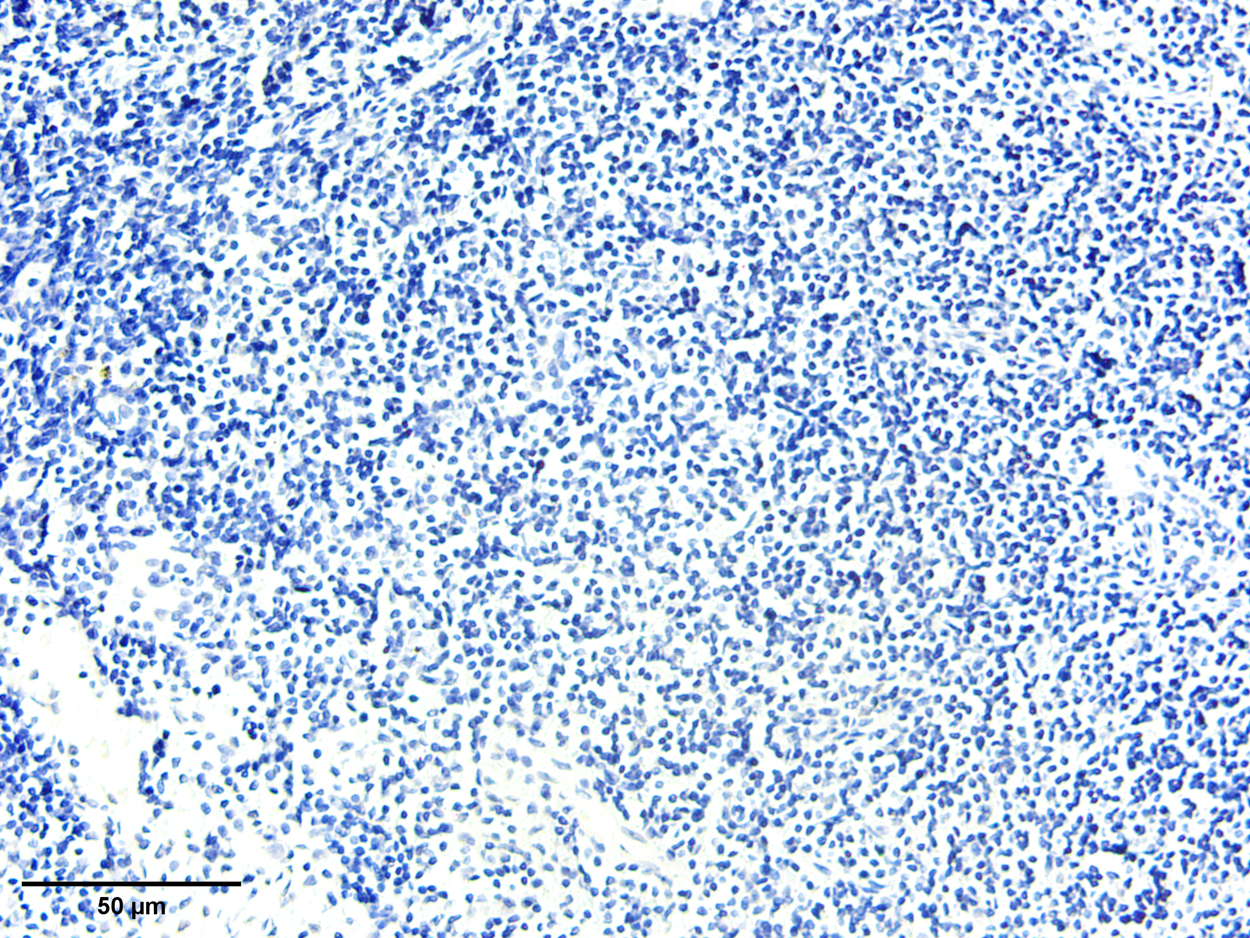

Supplement: Supplementary file 2 [file Data_Sheet_1.zip › 300dpi/Figure S2 L.JPG]

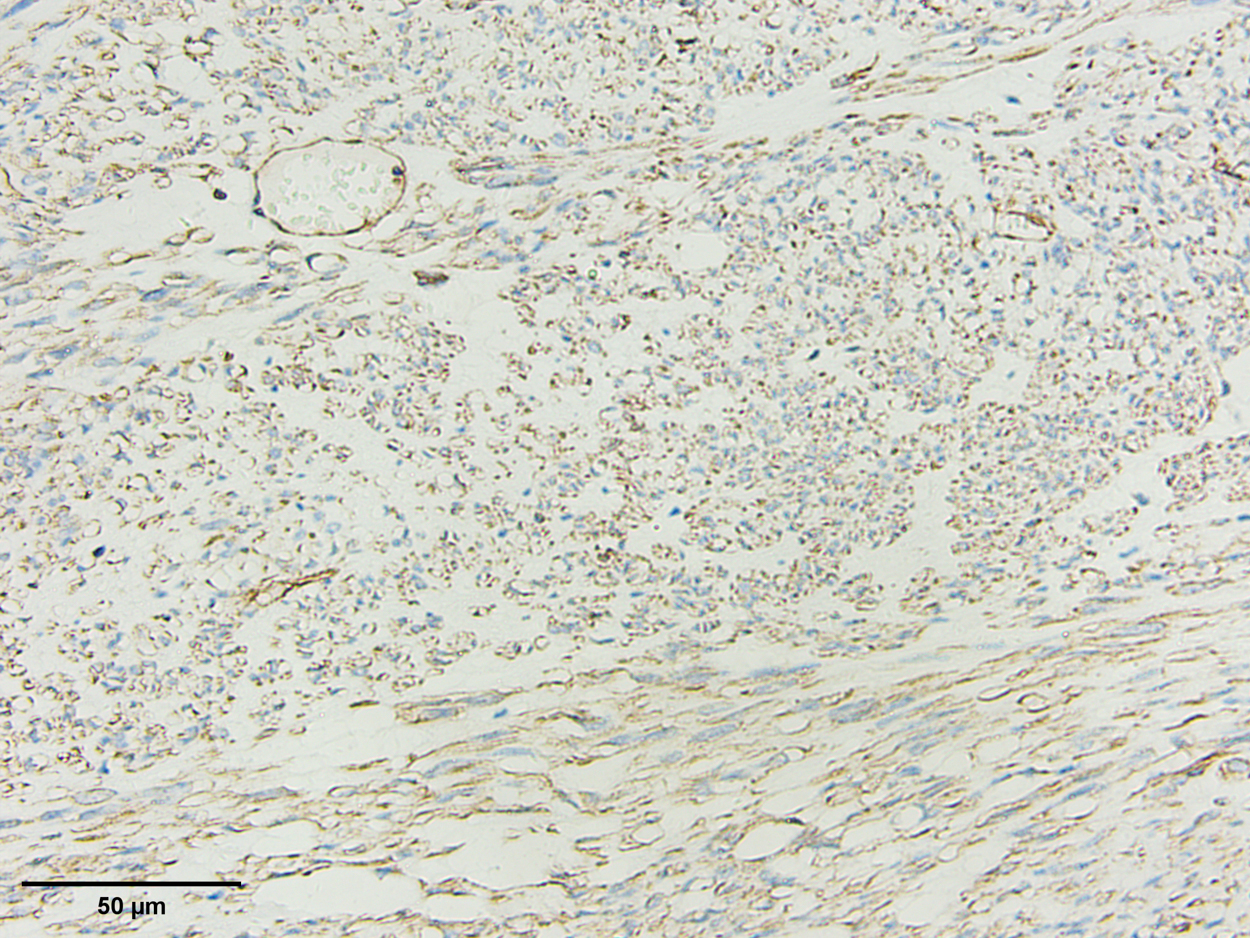

Supplement: Supplementary file 2 [file Data_Sheet_1.zip › 300dpi/Figure S2 M.JPG]

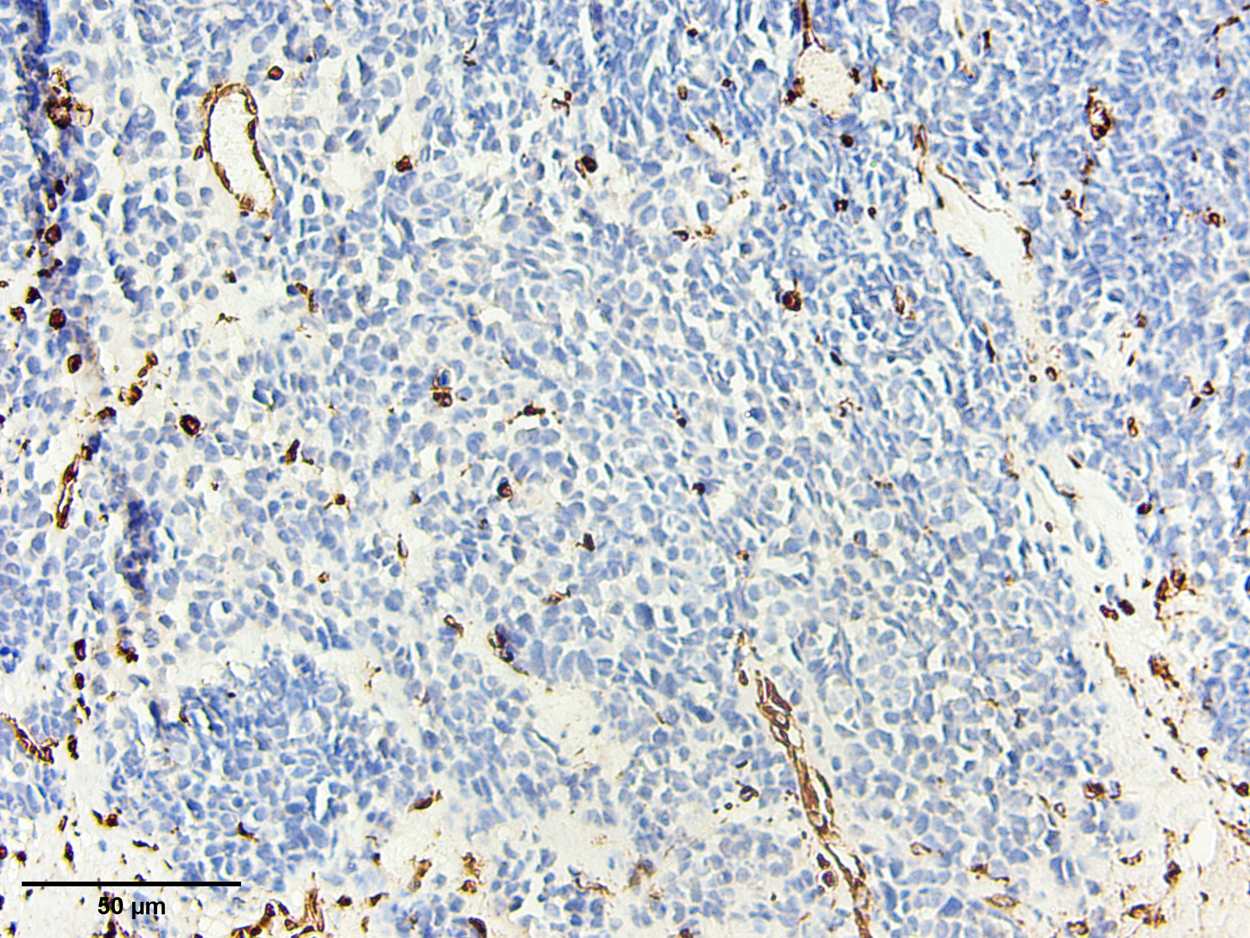

Supplement: Supplementary file 2 [file Data_Sheet_1.zip › 300dpi/Figure S2 N.JPG]
